# Cellular and molecular mechanisms breaking immune tolerance in inborn errors of immunity

**DOI:** 10.1038/s41423-020-00626-z

**Published:** 2021-04-01

**Authors:** Georgios Sogkas, Faranaz Atschekzei, Ignatius Ryan Adriawan, Natalia Dubrowinskaja, Torsten Witte, Reinhold Ernst Schmidt

**Affiliations:** 1 Department of Rheumatology and Immunology, Hannover Medical School, Hanover, Germany; 2 Hannover Medical School, Cluster of Excellence RESIST (EXC 2155), Hanover, Germany

**Keywords:** Inborn errors of immunity, Primary immunodeficiencies, Autoimmunity, Rheumatic diseases, Biomarkers, Diagnostic markers

## Abstract

In addition to susceptibility to infections, conventional primary immunodeficiency disorders (PIDs) and inborn errors of immunity (IEI) can cause immune dysregulation, manifesting as lymphoproliferative and/or autoimmune disease. Autoimmunity can be the prominent phenotype of PIDs and commonly includes cytopenias and rheumatological diseases, such as arthritis, systemic lupus erythematosus (SLE), and Sjogren’s syndrome (SjS). Recent advances in understanding the genetic basis of systemic autoimmune diseases and PIDs suggest an at least partially shared genetic background and therefore common pathogenic mechanisms. Here, we explore the interconnected pathogenic pathways of autoimmunity and primary immunodeficiency, highlighting the mechanisms breaking the different layers of immune tolerance to self-antigens in selected IEI.

## Introduction

Autoreactivity may to some extent be physiological, participating in the positive selection of lymphocytes and homeostasis of the immune system, and autoreactivity can be traced in healthy individuals as circulating autoantibodies and minor lymphocytic tissue infiltrates.^1^ In contrast, aberrant responses to self-antigens underlie more than 80 inflammatory conditions, defined as autoimmune diseases. Common autoimmune diseases, such as rheumatoid arthritis (RA), Sjogren’s syndrome (SjS), and systemic lupus erythematosus (SLE), appear to have a polygenic nature and are often the consequence of a pathogenic interplay between environmental and genetic factors.^2–5^

The term human inborn errors of immunity (IEI) is synonymous for primary immunodeficiency disorders (PIDs) and covers disorders with diverse clinical manifestations, ranging from susceptibility to infections to immune dysregulation and malignancy. As these disorders are caused by monogenic germline mutations, the term IEI highlights the increasingly identified genetic background of PIDs. To date, more than 430 monogenic traits falling under IEI have been reported.^6^ Not all mutations within genes linked to an IEI are pathogenic. The localization and severity of a mutation within a particular gene determines the resulting molecular dysfunction and the consequent aberration of immunity and/or immune tolerance.^7^ Further, the incomplete penetrance and variable expressivity of certain mutations reported as disease-causing call into question the monogenic etiology of IEI and the definite division between monogenic and poly- or oligogenic PIDs and suggest the strong influence of additional genetic and/or epigenetic modifiers.^7^

Although the terms autoimmunity and immunodeficiency appear contradictory, PIDs, even combined immunodeficiencies (CIDs), can manifest with autoimmunity, which can be their prominent phenotype.^8,9^ Interestingly, genetic variants reported in the context of IEI as disease causing, have been identified among patients with clear rheumatic phenotypes.^10^ Further, genes linked to IEI have been identified as risk genes in autoimmune rheumatic diseases.^9–12^ Such genes at the crossroads of autoimmunity and immunodeficiency are involved in immune checkpoint pathways, antigen receptor or cytokine signaling and, especially, type I interferon responses (Table 1). In addition to the evidence on a shared genetic basis of autoimmunity and immunodeficiency, the idea of the continuum of immunological diseases with sheer immunodeficiency or autoimmunity phenotypes representing two extremes of overlapping phenotypes caused by interconnected pathomechanisms is supplemented through the increasing identification of autoantibody-induced susceptibility to infection.^13,14^ Certain anti-cytokine autoantibodies have been reported to cause susceptibility to infections mimicking IEI. For example, autoantibodies to IFNγ can cause susceptibility to mycobacterial disease,^15,16^ autoantibodies to GM-CSF can induce susceptibility to infections with *Aspergillus* or *Cryptococcus* species^17–19^ and autoantibodies against type I interferons have been recently identified to underlie life-threatening COVID-19 pneumonia.^20^

**Table 1:** Genes identified as conferring susceptibility to systemic rheumatic autoimmune diseases through genome-wide association studies (GWASs) whose variations have been reported to underlie inborn errors of immunity (IEI)

Gene	PID	Associated rheumatic disease in GWASs	Reference	
			(Rheumatic disease)	(PID)
***ATM***	Ataxia-telangiectasia	RA	^238^	^239^
***BACH2***	BRIDA/BACH2 insufficiency	RA SLE	^240,241^	^71^
***CTLA-4***	CTLA-4 insufficiency (CID/CVID-like disorder)	RA	^242^	^56^
***DEF6***	DEF6 deficiency (CID/CVID-like disorder)	SLE	^243^	^65^
***IL2RA***	CD25 deficiency (IPEX-like syndrome)	RA	^244^	^67^
***IFNGR2***	IFNγR2 deficiency or partial deficiency (MSMD)	SLE RA	^238,245^	^246,247^
***IRF4***	IRF4 deficiency (CID)	RA	^248^	^93^
***IRF7***	IRF7 deficiency	SLE	^249^	^250^
***NCF1***	CGD	SjS SLE RA	^251,252^	^253^
***NFKBIA***	IκBα gain-of-function syndrome	psoriasis (SpA)	^254^	^215^
***TNFRSF13C (BAFF-R)***	BAFF receptor deficiency	SjS	^255^	^256^
***TYK2***	TYK2 deficiency	SpA	^257^	^258^

*BRIDA* BACH2-related immunodeficiency and autoimmunity, *CGD* chronic, granulomatous disease, *CID* combined immunodeficiency, *CTLA-4* cytotoxic T-lymphocyte-associated protein 4, *CVID* common variable immunodeficiency, *DEF6* differentially expressed in FDCP 6 homolog, *GWAS* genome-wide association study, *IFNγR* interferon γ receptor, *IPEX* Immunodysregulation, polyendocrinopathy, enteropathy, and X-linked, *IRF* Interferon regulatory factor, *MSMD* Mendelian susceptibility to mycobacterial disease, *PID* primary immunodeficiency disorder, *RA* rheumatoid arthritis, *SjS* Sjögren’s syndrome, *SLE* systemic lupus erythematosus, *SpA* spondyloarthritis

In the present review, we discuss the genetic basis of autoimmunity in PIDs. Further, we explore how particular genetic defects linked to the currently known IEI break the different layers of immune tolerance to self-antigens. Our review is by no means an exhaustive investigation of the origins of autoimmunity in all known IEI. Instead, we highlight the diverse pathophysiological pathways underlying autoimmunity and their relevance for selected IEI.

## Ineffective central tolerance

**Fig. 1:**
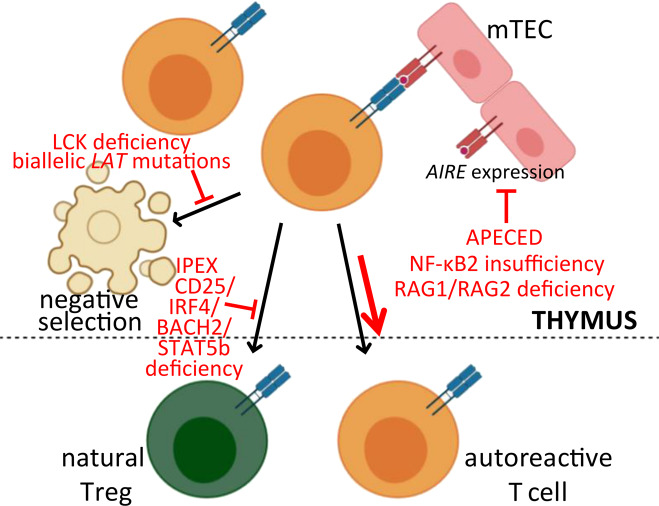
Inborn errors of immunity (IEI) impairing the induction of central T-cell tolerance. AIRE medullary thymic epithelial cells (mTECs) express an array of tissue-specific antigens. Autoreactive T-cell precursors recognizing self-antigens with relatively high avidity undergo clonal deletion (negative selection) or differentiate into natural regulator cells (Tregs). However, some autoreactive T cells skip central tolerance and escape the thymus. Monogenic immunodeficiency disorders affect antigen presentation by mTECs, clonal deletion or T-cell differentiation into natural Tregs; monogenic disorders and the level at which they impair or likely impair central T-cell tolerance are highlighted in red [APECED autoimmune polyendocrinopathy, candidiasis and ectodermal dystrophy, IPEX immunodysregulation, polyendocrinopathy, enteropathy and X-linked]

Random recombination events during thymic T-cell development yield a broad T-cell repertoire, including a large proportion of autoreactive thymocytes.^21^ Central T-cell tolerance is achieved through the mechanism of negative selection, whereby thymocytes recognizing self-antigens displayed on MHC molecules of medullary epithelial cells (mTECs) or thymic dendritic cells (DCs) with higher affinity undergo clonal deletion (Fig. 1). An alternative fate for autoreactive thymocytes is their differentiation into natural or constitutive CD4^+^CD25^+^Foxp3^+^ regulatory T cells (Tregs), which are able to suppress the induction and activation of effector T cells, preventing, or regulating immune responses.^22,23^ Despite their thymic origin, natural Tregs, together with adaptive or inducible Tregs, which have an extrathymic origin, are considered mechanisms of peripheral tolerance and will be discussed as such in this review.

### Autoimmune polyendocrinopathy-candidiasis-ectodermal dystrophy

Autoimmune polyendocrinopathy-candidiasis-ectodermal dystrophy (APECED, also known as autoimmune polyglandular syndrome type 1) is a rare multiorgan autoimmune disease caused by biallelic loss-of-function mutations in the gene encoding the autoimmune regulator (AIRE).^24,25^ AIRE is a transcription regulator orchestrating the expression of tissue-specific antigens (TSAs) by mTECs. As discussed previously, the latter is crucial for the induction of central tolerance through the process of negative selection. Autoimmunity in APECED commonly results in polyendocrinopathy but can affect nearly all organs. Chronic mucocutaneous candidiasis is the major infectious manifestation of APECED. A minority of patients display recurrent herpetic infections (HSV and VZV). Further, some patients are susceptible to infections with encapsulated bacteria, as they develop asplenia. Chronic mucocutaneous candidiasis in APECED is associated with the presence of neutralizing autoantibodies against Th17 cytokines (IL-17A, IL-17F, and IL-22), which suggests the autoimmune origin of immunodeficiency in this disorder.^26,27^

### DiGeorge syndrome

DiGeorge syndrome (or 22q11.2 deletion syndrome) is the consequence of disturbed development of the pharyngeal pouches, especially of the third and fourth pouches, which causes thymic hypoplasia or aplasia.^28^ Residual thymic function defines the degree of T-cell deficiency, which can range from a severe combined immunodeficiency (SCID)-like phenotype with the absence of T cells to normal T-cell counts and function. DiGeorge syndrome results in a relatively high prevalence (~8% of patients) of autoimmunity, most commonly manifesting as autoimmune cytopenia. Considering that DiGeorge syndrome is the consequence of disturbed development of the thymus, autoimmunity is thought to stem from defects in central tolerance. However, unlike patients with APECED, most DiGeorge syndrome patients display a single or two autoimmune diseases, and autoimmune manifestations have a later onset, precluding major defects in negative selection.^29^ Low numbers of Tregs may be the consequence of reduced thymic generation of natural Tregs and may also account for autoimmunity in DiGeorge syndrome, though Treg counts did not correlate with autoimmune disease.^30,31^

### NF-κB2 insufficiency

PID due to damaging monoallelic variants in *NFKB2* leads to immunodeficiency characterized by recurrent respiratory tract infections and failed control of herpesviruses.^32^ In addition to immunodeficiency, the vast majority (~80%) of patients display at least one autoimmune manifestation, including autoimmune cytopenias, arthritis, and alopecia. Studies on NF-κB2-deficient mice revealed the significance of the alternative NF-κB pathway in the induction of central tolerance, as NF-κB2 controls AIRE expression and is required for the development of mTECs.^33,34^ Therefore, the breakdown of tolerance in NF-κB2 deficiency mimics the pathophysiology of APECED. Mice harboring the *Lym1* mutation in *Nfkb2*, which prevents the processing of the precursor protein of NF-κB2, displayed lung and liver autoimmune infiltrates associated with decreased thymic expression of Aire.^35^ Even in the context of haploinsufficiency, mice displayed similar though milder autoimmunity. These mouse-derived findings together with the phenotypic overlap between APECED and NF-κB2 insufficiency,^32^ suggest impaired induction in central tolerance as the main mechanism of autoimmunity. In addition, patients with *NFKB2* mutations display reduced Treg counts.^36,37^ This, together with the fact that mice with conditional deletion of *nfkb2* in Tregs develop lethal autoimmunity due to the impaired suppressive function of Tregs,^38^ suggests an additional Treg defect, which may be relevant in the pathogenesis of autoimmunity in NF-κB2 insufficiency.

### Omenn syndrome

Omenn syndrome is a genetically heterogeneous disorder commonly linked to biallelic mutations in recombinase activating gene 1 (*RAG1*) or recombinase activating gene 2 (*RAG2*).^39^ Mutations in other genes involved in somatic V(D)J recombination, the process defining the B-cell and T-cell receptor repertoire, can result in Omenn-syndrome-like disease with generalized dermatitis and lymphadenopathy-associated with oligoclonal T-cell expansion. Given its genetic background, Omenn syndrome highlights an impaired T-cell repertoire as a mechanism of autoimmunity in primary immunodeficiency. The expression of AIRE is reduced in the thymus of patients with Omenn syndrome, suggesting aberrations of central tolerance and negative selection.^40^ Decreased suppressive function of Tregs has been reported in patients with Omenn syndrome and may represent an alternative or additional pathomechanism compromising tolerance.^41^

## Regulatory T cells

**Fig. 2:**
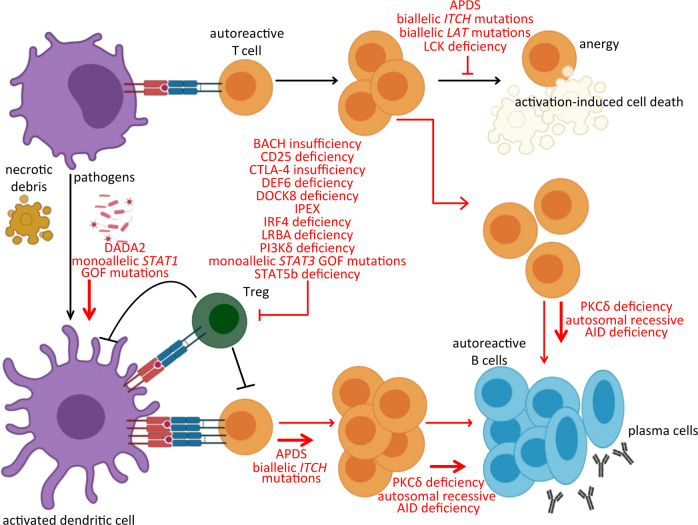
Inborn errors of immunity (IEI) impairing the induction of peripheral tolerance. In the absence of adequate costimulation, the recognition of self-antigens displayed by immature dendritic cells has a tolerogenic outcome, resulting in anergy or clonal deletion. Tissue damage, however, can break the ‘immune privilege’ at the tissue or subcellular level, facilitating the presentation of self-antigens. If this happens in a milieu supporting dendritic cell activation, such as in the presence of uncontrolled proinflammatory cytokine signaling or in the context of persistent infection or Treg dysfunction, an autoimmune T-cell response can be primed and result in the activation of autoreactive B cells. The source of B-cell autoreactivity is either aberrant central B-cell tolerance or de novo generation in the context of a germinal center reaction (not shown). Monogenic immunodeficiency disorders affect peripheral tolerance by enhancing the capacity of antigen-presenting cells to prime T cells, by compromising Treg function or reducing their counts, by enhancing antigen receptor-mediated activation of lymphocytes and/or by impairing tolerogenic aspects of antigen receptor signaling; monogenic disorders and the level at which they impair peripheral tolerance are highlighted in red [GOF gain-of-function, APDS activated PI3Kδ syndrome, DADA2 deficiency of ADA2]

Despite the dominant tolerogenic function of the thymus, the induction of central tolerance is incomplete.^42^ Significant numbers of autoreactive T cells that could trigger autoimmunity are detected in the circulation of healthy individuals, highlighting the significance of the mechanisms inducing peripheral tolerance (Fig. 2).^43,44^ Tregs are a major group of immunosuppressive T cells that play an essential role in the maintenance of peripheral immune tolerance and the regulation of immune responses. Treg dysfunction has been reported for a variety of autoimmune diseases, including type 1 diabetes (T1D), RA, multiple sclerosis (MS), SLE, myasthenia gravis, and systemic sclerosis.^45^ Forkhead box P3 (FOXP3) is indispensable for their development and function, and the most definitive evidence on the role of both FOXP3 and Tregs in the maintenance of tolerance came from genetic studies on Scurfy (Sf) mice, which display fatal multiorgan autoimmunity as a consequence of mutations in *Foxp3*.^46^

Monogenic diseases resulting in Treg deficiency (Table 2) highlight the dominant tolerogenic role of Tregs. The prototype of so-called Tregopathies is immunodysregulation, polyendocrinopathy, enteropathy, and X-linked (IPEX) syndrome, which is caused by biallelic loss-of-function mutations in *FOXP3*; therefore, patients with IPEX syndrome are the human equivalent of Scurfy mice.^46,47^ To date, 111 different *FOXP3* mutations, located throughout the *FOXP3* gene, have been reported in patients with IPEX syndrome.^48^ No clear phenotype*-*genotype correlation has been identified, and those patients typically display a triad of clinical manifestations consisting of early-onset severe enteropathy, T1D, and dermatitis.^49^ Other autoimmune manifestations include autoimmune cytopenias, arthritis, autoimmune thyroiditis, nephropathy, and hepatitis.

**Table 2:** Primary immunodeficiency disorders resulting in immune dysregulation primarily associated with regulatory T-cell dysfunction

Mutated gene (protein)/locus	Inheritance	Immunodeficiency/infectious manifestations	Autoimmune/lymphoproliferative manifestations	References
***BACH2*** **(BACH2)/6q15**	AD	Variable hypogammaglobulinemia (but also hypergammaglobulinemia), reduced memory and class-switched memory B cells	IBD, lymphadenopathy, interstitial lung disease, autoantibodies	^71^
***CTLA-4*** **(CTLA-4)/2q33.2**	AD	Hypogammaglobulinemia (84%), herpes infection (30%), bacterial infections (30%), fungal infection (18%)	Gastrointestinal involvement (59%), cytopenia (59%), endocrinopathy (33%), arthritis (14%), renal (12%) and liver involvement (12%)	^56^
***DEF6*** **(DEF6/IBP/SLAT)/6p21.31**	AR	Variable hypogammaglobulinemia, low class-switched memory B cells, recurrent bacterial infections, herpes infection	IBD, AIHA, detection of autoantibodies, such as ANCA, ANA and anti-cardiolipin	^65^
***DOCK8*** **(DOCK8)/9p24.3**	AR	Elevated IgE, hypogammaglobulinemia in some patients, failure of long-lived antibody responses to vaccines, T-cell lymphopenia, decreased numbers of naïve T cells, sinopulmonary infections, bronchiectasis, viral infections (CMV, EBV, HSV, HPV), PML, abscesses, fungal infections, including mucocutaneous candidiasis	Autoimmune cytopenia, vasculitis, SLE, hypothyroidism, arthritis, IBD, IPEX-like disease	^87,88^
***IL2RA*** **(CD25)/10p15.1**	AR	Upper and lower respiratory tract infections, persistent EBV and CMV infections	IPEX-like disease (enteropathy, eczema, endocrinopathy, autoimmune cytopenias, lymphadenopathy, organomegaly)	^67,68^
***IRF4*** **(IRF4)/6p25.3**	AR	Agammaglobulinemia, thrush, bronchopneumonia	Autoimmune polyendocrinopathy	^93^
***LRBA*** **(LRBA)/4q31.3**	AR	Recurrent upper and/or lower respiratory tract infections/hypogammaglobulinemia with reduced class-switched memory B cells in the majority of patients	Autoimmune cytopenia, ILD, autoimmune hepatitis, alopecia, enteropathy, lymphadenopathy, organomegaly	^60^
***PIK3CD*** **(p110δ**^a^**)/1p36.22**	AR^b^	Hypogammaglobulinemia (all reported patients), upper and/or lower respiratory infections (all reported patients)	IBD (50%), arthritis, ITP, psoriasis, autoimmune hepatitis	^94^
***STAT3*** **(STAT3)/17q21.2**	AD^c^	Hypogammaglobulinemia, low class-switched memory B cells and low naïve CD4^+^ T cells, bronchopulmonary infections (>40%), bronchiectasis (~10%), *Candida* infections	Lymphoproliferation (>60%), autoimmune cytopenia (>60%), IBD (~50%), ILD (~40%)	^82^
***STAT5b*** **(STAT5b)/17q21.2**	AR	Variable degree of immunodeficiency, including recurrent respiratory tract and cutaneous infections Mild lymphopenia (reduced T cells, B cells and/or NK cells) reduced T-cell proliferation to mitogens and antigens	IPEX-like disease (enteropathy, eczema, endocrinopathy), alopecia, interstitial pneumonitis	^76^

*AD* autosomal dominant, *AIHA* autoimmune hemolytic anemia, *ANA* antinuclear antibodies, *ANCA* anti-neutrophil cytoplasmic antibodies, *AR* autosomal recessive, *BCG* bacillus Calmette-Guérin, *CMV* cytomegalovirus, *EBV* Epstein–Barr virus, *HSV* herpes simplex virus, *HPV* human papillomavirus, *IBD* inflammatory bowel disease, *ILD* interstitial lung disease, *IPEX* immunodysregulation, polyendocrinopathy, enteropathy, and X-linked, *ITP* immune thrombocytopenic purpura, *PML* progressive multifocal leukoencephalopathy, *SLE* systemic lupus erythematosus, *VZV* varicella zoster virus

^a^Phosphatidylinositol 4,5-bisphosphate 3-kinase catalytic subunit

^b^Loss-of-function *PIK3CD* variants

^c^Gain-of-function *STAT3* variants

### CTLA-4 insufficiency

Cytotoxic T-lymphocyte protein 4 (CTLA-4) is a transmembrane protein expressed mainly by T cells.^50^ It interacts with CD80 and CD86 on the surface of antigen-presenting cells (APCs) and functions as a coinhibitory molecule. This interaction has a higher affinity than that of the costimulatory molecule CD28 with CD80 and CD86, counteracting its activating effect.^51^ Regulatory T cells constitutively express CTLA-4, whereas its expression on the plasma membrane of conventional T cells follows their activation. The balance between CD28 and CTLA-4 signaling is critical for the outcome of T-cell interactions with APCs, which may lead to either an effective adaptive immune response or a primarily tolerogenic response. CTLA-4-deficient mice die of early-onset multiorgan autoimmunity, demonstrating the dominant tolerogenic function of CTLA-4.^52^ In contrast, heterozygosity for the *Ctla-4* knockout mutation has been reported to result in a normal phenotype.^53^ Therefore, it was not until the description of human CTLA-4 insufficiency that it became clear that not just the presence of CTLA-4 but also the amount of its expression by T cells is critical for immune homeostasis and the maintenance of immune tolerance.^54^ CTLA-4 insufficiency in humans compromises the function of Tregs, resulting in lymphoproliferation and autoimmunity.^55^ To date, more than 50 heterozygous germline mutations have been reported to account for CTLA-4 insufficiency in more than 130 patients.^54^ Autoimmune manifestations of CTLA-4 insufficiency resemble those of IPEX syndrome, including enteropathy, autoimmune cytopenias, arthritis, and endocrinopathy.^56^ However, incomplete penetrance and variable expressivity of genetic variants causing CTLA-4 insufficiency question the strictly monogenic origin of immune dysregulation in this disorder and suggest the influence of additional genetic or epigenetic modifiers and environmental factors.

### LRBA deficiency

Lipopolysaccharide-responsive and beige-like anchor protein (LRBA) deficiency, due to germline biallelic mutations in *LRBA*, phenocopies CTLA-4 insufficiency.^57,58^ The explanation for this is the fact that LRBA controls intracellular trafficking of CTLA-4, and a loss of LRBA expression results in reduced expression and mobilization of CTLA-4 on the surface of Tregs. In addition to the impaired suppressive function of Tregs, which is at least partially explained by the reduction in CTLA-4 expression,^59^ the majority of LRBA-deficient patients display reduced Treg counts.^60^ Autoimmunity is reported to be the most common manifestation of LRBA deficiency and includes enteropathy, autoimmune cytopenias, endocrinopathy, interstitial lung disease, and/or autoimmune hepatitis. The CTLA-4-Fc fusion molecule (abatacept) has been reported to ameliorate manifestations of LRBA deficiency, such as enteropathy and lung disease, which strongly suggests that a loss of proper expression of CTLA-4 is a major pathogenic mechanism underlying immune dysregulation in these patients.^57,61^

### DEF6 deficiency

The differentially expressed in FDCP 6 homolog (DEF6), also known as IRF4 binding protein (IRF4BP), is a guanine nucleotide exchange factor (GEF) that transmits TCR signaling.^62^ DEF6 activates small GTPases, promoting calcium signaling and T-cell adhesion, and it is involved in the formation of immunological synapses as well as in T-cell differentiation and proliferation. Def6 deficiency in murine autoimmunity appears to depend on the genetic background and/or the employed model of autoimmune disease.^63,64^ The recent discovery of human DEF6 deficiency, which manifests as systemic autoimmunity and lymphoproliferation, revealed the homeostatic role of DEF6 in the human immune system.^65^ Similar to LRBA deficiency, DEF6 deficiency causes a CTLA-4 trafficking defect, resulting in reduced stimulation-induced CTLA-4 expression by Tregs. The latter, together with the phenotypic overlap between DEF6 deficiency and LRBA deficiency or CTLA-4 insufficiency and the reported therapeutic efficacy of CTLA-4-Ig therapy (abatacept) in treating autoimmunity, suggest that a loss of proper trafficking of CTLA-4 is a major mechanism of immune dysregulation in DEF6 deficiency.

### CD25 deficiency

CD25 is the α chain of the interleukin 2 receptor (IL-2Rα), and it is constitutively expressed by natural Tregs.^66^ CD25 confers high-affinity binding of interleukin 2 (IL-2) to the trimeric IL-2 receptor, additionally consisting of a β chain (CD122, IL-2Rβ) and the common γ chain (CD 132, IL-2Rγ_c_), which both mediate receptor signaling. CD25 deficiency results in an IPEX-syndrome-like phenotype, with early-onset autoimmunity and lymphoproliferation but also severe immunodeficiency, causing chronic viral infections as well as fungal and bacterial infections.^67,68^ In all identified patients, CD25 expression was abrogated, which makes the isolation of Tregs and the evaluation of the impact of CD25 deficiency on Treg function difficult.^47^ However, the study of Tregs from patients with CD25 deficiency as CD4^+^CD45RO^+^TIGIT^+^CD127^low^ T cells demonstrated both their reduced frequency and their suppressive function.^69^ The latter has been suggested to be mainly because CD25 deficiency deprives Tregs from their capacity to consume IL-2.^67^ Consistent with this, adoptive transfer of CD25^+^ Tregs into CD25-deficient mice drastically decreased serum levels of IL-2 and restored abnormalities in peripheral lymphocyte subsets, highlighting the homeostatic role of IL-2 consumption by Tregs in the immune system.^70^

### BACH2 insufficiency

The broad complex-tramtrack-bric a brac and Cap'n'collar homology 2 (BACH2) is a basic leucine zipper domain transcription factor involved in the maturation and differentiation of both T cells and B cells.^71^ BACH2-regulated gene expression promotes Treg development at the expense of effector T-cell differentiation, as demonstrated in mice lacking BACH2.^71,72^ Further, it promotes the survival of Tregs, regulates their activation, and is required for the development and maintenance of tissue-resident Tregs, especially in the gastrointestinal tract.^73^ BACH2 insufficiency has been reported in three patients from two unrelated families who displayed benign lymphoproliferation, enteropathy, and recurrent respiratory tract infections.^71^ The identification of low counts of peripheral blood Tregs in these patients is consistent with previous reports on BACH2-deficient mice and suggests a similar role of BACH2 in human Treg development.

### STAT5b deficiency

Signal transducer and activator of transcription 5b (STAT5b) deficiency results in a combined immunodeficiency with severe growth delay and immune dysregulation commonly manifesting as interstitial lung disease.^43,74^ Growth delay reflects the involvement of STAT5b in growth hormone signal transduction. In lymphocytes, STAT5 associates with the IL-2Rβ chain of IL-2R, and immune dysregulation is the consequence of the loss of IL-2R-mediated STAT5 activation, which is required for sustained FOXP3 expression and the development of natural Tregs.^75^ Further, STAT5 upregulates IL-2Rα, activating a positive feedback loop, which enhances Treg responsiveness to IL-2. Similar to CD25 deficiency, STAT5b-deficient patients display an IPEX-syndrome-like phenotype and have reduced circulating Treg counts with compromised suppressor function.^74,76,77^

### STAT3 gain of function

Signal transducer and activator of transcription 3 (STAT3) is a transcription factor that transmits the signaling of several cytokine receptors, such as those of IL-2, IL − 6, IL − 10, IL − 12, IL − 15, and IL − 23, controlling T-cell activation and differentiation.^78^ *STAT3* loss-of-function mutations account for autosomal-dominant hyper-IgE syndrome (HIES), which is a primary immunodeficiency characterized by very high serum IgE levels, atopic eczema, bacterial and fungal infections, and various nonimmune developmental manifestations.^79,80^ More recently, germline heterozygous gain-of-function mutations in *STAT3* were reported to account for early-onset organ-specific autoimmunity, eczema, and short stature.^81^ Enteropathy, autoimmune cytopenias, interstitial lung disease, and lymphoproliferative and infectious manifestations are also common in patients with STAT3 gain of function.^82^ The phenotypic variability of this syndrome can be at least partially explained through the variable functional impact of the different disease-causing variants, which differentially affect baseline and induced activation of STAT3. In particular, in their recent study, Jägle et al. evaluated the biological impact of 17 different *STAT3* gain-of-function variants and proposed their clustering in three main groups: variants causing enhanced basal transcriptional activity of STAT3 as well as altered inducible STAT3 phosphorylation, variants predominantly affecting inducible activation of STAT3 and those resulting in enhanced DNA binding of STAT3. *STAT3* variants, both those enhancing baseline transcriptional activity and those affecting inducible phosphorylation of STAT3, have been reported to more consistently cause immune dysregulation, displaying the highest penetrance for autoimmune and/or lymphoproliferative disease. The majority of *STAT3* gain-of-function mutations resulted in reduced peripheral Treg counts. Further, reduced suppressive function of Tregs has been reported for some patients.^83,84^ Mechanistically, STAT3 activation results in the upregulation of secretion of cytokine signaling 3 (SOCS3).^85,86^ The latter inhibits STAT5 activation, which—as discussed above—induces FOXP3 and CD25 expression. Therefore, the potentiation of this signaling loop through STAT3 hyperactivation results in decreased differentiation and function of Tregs, likely accounting for autoimmunity in patients with STAT3 gain of function.

### DOCK8 deficiency

Dedicator of cytokinesis 8 (DOCK8) is a GEF that is highly expressed in lymphocytes.^87^ DOCK proteins activate small guanine triphosphate binding proteins (GTPases), such as RAC and CDC42, affecting the actin cytoskeleton. Germline biallelic loss-of-function mutations in *DOCK8* cause an autosomal-recessive form of HIES. In comparison to other monogenic PIDs, mutations in *DOCK8* are common deletions. An explanation for this is the high frequency of repetitive sequence elements within and around *DOCK8*. In addition to infectious manifestations, including candidiasis, recurrent respiratory tract infections, and persistent cutaneous viral infections, DOCK8 deficiency has been associated with immune dysregulation manifesting as autoimmunity and atopy.^87,88^ Autoimmunity in DOCK8 deficiency has been reported to manifest as vasculitis, autoimmune hepatitis, or IPEX-syndrome-like disease. The identification of an increased proportion of autoreactive B cells within the compartment of mature naïve B cells in DOCK8-deficient patients suggests a defect in peripheral tolerance.^89^ Incomplete induction of peripheral tolerance and autoimmunity in DOCK8 deficiency reflects the involvement of DOCK8 in Treg homeostasis and function. Consistent with the aforementioned is the fact that patients with DOCK8 deficiency display low Treg counts with reduced suppressive function, though the exact mechanism accounting for this remains unknown.^89^

### IRF4 deficiency

Interferon regulatory factor 4 (IRF4) is involved in T helper cell polarization and is required for the effector function of Tregs as it controls IL-10 and ICOS expression.^90^ Further, IRF4 expression by thymic epithelial cells is critical for efficient priming of natural Tregs.^91^ IRF4-deficient mice display progressive immune dysregulation and severe hypogammaglobulinemia.^92^ The first description of human IRF4 deficiency due to a germline homozygous splice acceptor site mutation in *IRF4* has been recently reported and resembles the phenotype of IRF4-deficient mice.^93^ This patient displayed agammaglobulinemia and early-onset autoimmunity manifesting as polyendocrinopathy, including T1D, and displayed a low Treg count. She additionally suffered from eczema and died at the age of 2 years after allogeneic hematopoietic stem cell transplantation.

### PI3Kδ deficiency

The relatively recently characterized IEI due to germline biallelic loss-of-function mutations in *PIK3CD* or *PIK3R1* results in immunodeficiency, primarily due to severe hypogammaglobulinemia, as well as autoimmunity in the form of arthritis, psoriasis, and inflammatory bowel disease (IBD), most likely as a consequence of the compromised suppressive function of Tregs.^94–96^ In particular, mouse studies have suggested the involvement of PI3Kδ in the development and suppressive function of Tregs.^97–99^ However, as mentioned previously, all tested patients with biallelic mutations in *PIC3CD* or *PIK3R1*, resulting in a loss of PI3Kδ activity, had adequate Treg counts in their peripheral blood. The fact that the PI3Kδ-specific inhibitor idelalisib compromises the suppressive function of Tregs in vitro and ex vivo in patients with chronic lymphocytic leukemia,^100^ together with the fact that common side effects of idelalisib treatment resemble the inflammatory manifestations of a loss of PI3Kδ activity,^101^ strongly suggest immune dysregulation owing to the inadequate suppressive function of Tregs in this IEI.^94^

## Aberrant T-cell receptor (TCR) signaling

TCR signaling controls the development and peripheral responses of T cells, ensuring both effective immunity and immune tolerance.^102^ TCR signaling defects could compromise central tolerance by affecting negative selection of autoreactive T cells. In addition, aberrant TCR signaling can affect the development and function of regulatory T cells, impairing the induction of peripheral tolerance. Finally, mutations in molecules involved in the TCR signaling cascade may result in autoimmunity by hyperactivating autoreactive T cells or depriving TCR signaling from tolerogenic aspects, such as the induction of anergy or activation-induced cell death.

### ORAI1/STIM1 deficiency

The pathway of store-operated calcium entry (SOCE) is the main mode of calcium influx in immune cells and is involved in TCR, B-cell receptor (BCR), Fc gamma receptor (FcγR), and Fc epsilon receptor (FcεR) signaling as well as downstream effector responses.^103,104^ SOCE is activated in response to the depletion of intracellular stores of calcium, particularly of the endoplasmic reticulum. Stromal interaction molecule 1 (STIM1) has been identified as the major calcium sensor in the ER membrane that activates calcium channels in the plasma membrane upon calcium store release. Orai1 is the major calcium channel activated by STIM1 in most immune cells, especially in B cells and T cells.

Biallelic loss-of-function mutations in *STIM1* and *ORAI1*, leading to STIM1 or ORAI1 deficiency, respectively, manifest with combined immunodeficiency and autoimmunity in the form of autoimmune hemolytic anemia and thrombocytopenia.^104^ Patients with STIM1 deficiency, as well as those with ORAI1 deficiency, display reduced Treg counts.^105,106^ The role of store-operated calcium entry (SOCE) in the development of regulatory T cells has been demonstrated in mice with T-cell-targeted deletion of both STIM isoforms, which have reduced peripheral and tissue-resident Tregs and develop autoimmunity and lymphoproliferation.^107,108^ While a reduction in Treg counts could explain autoimmunity in those patients, even in the case of STIM1 deficiency, which is more consistently associated with immune dysregulation, patients do not display an IPEX-syndrome-like phenotype. Impaired negative selection may also contribute to autoimmunity, though the normal Vβ repertoire of TCRαβ^+^ T cells in patients with *STIM1* mutations suggests the absence of major defects in the induction of central tolerance.^109,110^ The recently reported regulatory function of STIM1 in controlling type I interferon responses^111^ could also be relevant in the pathogenesis of autoimmunity, at least in STIM1-deficient patients, and is consistent with the SLE-like autoantibody profile detected in these patients.^105^

### Activated phosphoinositide 3-kinase δ syndrome

Phosphoinositide 3-kinase delta (PI3Kδ) mediates signal transduction downstream of diverse immune receptors, including the TCR, the BCR, costimulatory molecules, and cytokine receptors.^94^ The hyperactivation of PI3Kδ due to monoallelic gain-of-function mutations in *PIK3CD* or due to monoallelic loss-of-function mutations in *PIK3R1* or *PTEN* results in immunodeficiency and immune dysregulation.^112,113^ In particular, the hyperactivation of PI3Kδ results in combined immunodeficiency, characterized by recurrent respiratory tract infections, bronchiectasis, mucocutaneous candidiasis, and susceptibility to viral infections, including failed control of herpesviruses.

Immune dysregulation as a consequence of the hyperactivation of PI3Kδ leads to benign lymphoproliferation, manifesting as lymphadenopathy, hepatomegaly, splenomegaly, and nodular lymphoid hyperplasia of mucosal surfaces as well as malignant lymphoproliferative disease and especially B-cell lymphomas. Further, patients with immune dysregulation due to the hyperactivation of PI3Kδ display a broad spectrum of autoimmune manifestations, including cytopenias, endocrinopathies, glomerulonephritis, and Sjögren’s syndrome. Immune dysregulation in patients with hyperactivated PI3Kδ is most likely the consequence of the hyperactivation of T cells.^94,112–114^ Studies on mice with hyperactivated PI3Kδ suggest defects in thymic negative selection but also ineffective peripheral tolerance due to resistance to activation-induced cell death and increased secretion of effector cytokines by T cells, which has also been demonstrated in patients with activated phosphoinositide 3-kinase δ syndrome (APDS).

### ITCH E3 ubiquitin ligase deficiency

The ubiquitination of TCRs reduces their expression and therefore regulates T-cell activation.^115^ Itchy E3 ubiquitin-protein ligase, referred to hereafter as Itch, is a key tolerogenic molecule involved in this process. In addition to TCR subunits, Itch is involved in the ubiquitination of molecules involved in proximal TCR signaling, such as the TCRζ chain and mitogen-activated protein kinase kinase1 (MEKK1). In addition, Itch regulates Notch, a transcription factor promoting TCR signaling. The dysregulation of TCR-Notch signaling can drive both lymphoproliferation and autoimmunity.^116^ Itch-deficient T cells have a lower activation threshold, which, in the case of autoreactive T cells, could enhance their activation and prevent tolerogenic mechanisms, such as their conversion into anergic T cells.^115^ In addition to controlling T-cell activation, evidence from *Itch*-knockout mice suggests the direct involvement of Itch in regulating proinflammatory NF-κB activation in response to TNF or IL-1.^117^ Biallelic mutations in itchy E3 protein ubiquitination ligase (*ITCH*) result in syndromic polyautoimmunity and immunodeficiency, highlighting the regulatory role of ubiquitination in controlling TCR signaling and regulating inflammation.^118,119^ Patients with Itch defects display recurrent infections, including bacterial sepsis; however, clinically more prominent is their craniofacial dysmorphism and immune dysregulation, resulting in hepatosplenomegaly, interstitial lung disease, enteropathy, autoimmune hepatitis, and endocrinopathy, including hypothyroidism and T1D.

### Biallelic loss-of-function mutations in *LAT*

Biallelic loss-of-function mutations in *LAT*, the gene encoding linker for activation of T cells (LAT), result in early-onset combined immunodeficiency and autoimmunity.^120^ In particular, those patients suffer from recurrent pneumonias, bronchiectasis, and herpesvirus infections, especially due to EBV or CMV. Immune dysregulation manifests as lymphadenopathy, splenomegaly, interstitial lung disease, and autoimmune cytopenia. Mechanistically, mutant LAT prevented TCR-downstream phosphorylation of phospholipase Cγ1 (PLCγ1). Mouse studies preceding the identification of the human PID due to biallelic LAT mutations have revealed the tolerogenic importance of TCR-induced docking PLCγ1 on LAT.^121^ In particular, mice harboring a homozygous mutation replacing the docking site of PLCγ1 on LAT, i.e., the tyrosine residue at position 136 of LAT, displayed lethal lupus-like autoimmunity. T cells from these mice displayed enhanced TCR-mediated activation. Further studies on these mice suggested that the uncoupling of LAT-PLCγ1 signaling may alter thymocyte selection, resulting in thymic release of autoreactive T cells, which would otherwise be eliminated through negative selection.^122^

### Biallelic loss-of-function mutations in *LCK*

Autosomal-recessive deficiency of lymphocyte-specific protein tyrosine kinase (LCK) has been reported in a girl with early-onset recurrent respiratory tract infections, including pneumonia complicated by pneumatocele.^123^ This patient additionally displayed neutrophilic panniculitis, polyserositis, and recession after the introduction of a TNF inhibitor and ITP, suggesting immune dysregulation. In addition to defects in thymic selection of T cells as a consequence of aberrant TCR signaling, LCK deficiency appears to result in resistance to activation-induced cell death and reduced Treg counts.

### Biallelic loss-of-function mutations in *CD3G*

Finally, autosomal-recessive CD3γ deficiency results in variable immunodeficiency, more commonly manifesting with hypogammaglobulinemia and recurrent respiratory tract infections.^124,125^ So far, all ten reported patients with CD3γ deficiency displayed autoimmune disease, most commonly manifesting as hypothyroidism and autoimmune cytopenias. Autoimmunity in CD3γ deficiency has been suggested to stem from thymic release of autoreactive T cells and in parallel Tregs with limited TCR diversity and reduced suppressive function.^124,125^

## Complement deficiencies causing autoimmunity

Complement deficiencies, especially those of early components of the complement activation cascade, can lead to both recurrent infections and autoimmunity.^12^ Early classic complement deficiencies (C1, C2, or C4) cause susceptibility to infections with encapsulated bacteria and are frequently associated with autoimmunity, especially SLE.^126,127^ A deficiency of C4 or any of the components of the C1 complex results more commonly in SLE than C2 deficiency (88% of patients with C1q deficiency, 75% in the case of C4 deficiency, 57% of those with C1s/r vs. 10% in the case of C2 deficiency). Further, SLE associated with C4 or one of the C1 complex deficiencies displays an earlier onset and more severe disease course than C2 deficiency-associated SLE.^126,128^ Defects in the lectin pathway of complement activation can also cause or contribute to the pathogenesis of autoimmunity. Mannose-binding lectin (MBL) deficiency has a prevalence of 5% and is the most common complement deficiency.^129^ A deficiency of MBL increases the risk of pyogenic infections, especially in infants. In addition, it is associated with an increased risk for autoimmunity.^130^ Mannan-binding lectin-associated serine protease 2 (MASP-2) activates the lectin pathway of complement, assuming a role analogous to that of C1s/C1r in classic activation of complement. In addition to susceptibility to infections, MASP-2 deficiency results in SLE-like autoimmunity.^131^ Early complement components are implicated in the effective clearance of self-antigens, exposing apoptotic cells and immune complexes.^12,130^ Defects in both of these processes can lead to autoimmunity or contribute to its pathogenesis.

## Immune dysregulation due to apoptosis defects

Lymphocyte expansion in the context of an adaptive immune response is controlled by apoptosis. Human defects in apoptosis leading to autoimmune lymphoproliferative disease (ALPS) highlight the homeostatic role of apoptosis-mediated clonal contraction.^132^

ALPS typically presents with chronic benign lymphoproliferation (lymphadenopathy, splenomegaly, and hepatomegaly), which may be accompanied by autoimmune cytopenias.^133,134^ Immune dysregulation in ALPS may also manifest with uveitis, thyroiditis, hepatitis, or SLE-like disease. Further, ALPS patients display an increased incidence of lymphoid malignancies. Susceptibility to infections is rare, mostly attributed to splenectomy or autoimmune neutropenia.^135^ Patients with Fas deficiency typically display marked elevations in CD4^-^CD8^-^ double-negative T cells.^136^ Other typical laboratory findings include elevated vitamin B12, IL-10, and soluble Fas ligand (FasL/CD95L) levels. Although polyclonal hypergammaglobulinemia is also relatively common in ALPS, less than 10% of patients display hypogammaglobulinemia, which in some cases may be associated with previous immunosuppressive treatment.^137^ The diagnosis of ALPS is based on clinical and laboratory findings (Table 3A).

**Table 3:** (A) Revised diagnostic criteria for the diagnosis of ALPS and (B) subtypes of ALPS^259^

(A) Revised diagnostic criteria for the diagnosis of ALPS
Required
1. Chronic (>6 months), nonmalignant, noninfectious lymphadenopathy or splenomegaly or both
2. Elevated CD3^+^, T-cell receptor αβ^+^, CD4^−^, CD8^−^ double-negative T cells (≥1.5% of total lymphocytes or 2.5% of CD3^+^ lymphocytes) in the setting of normal or elevated lymphocyte counts
Accessory
-Primary
1. Defective lymphocyte apoptosis (in 2 separate assays)
2. Somatic or germline pathogenic mutations in *FAS*, *FASL* or *CASP10*
-Secondary
1. Elevated plasma-soluble Fas ligand levels (>200 pg/ml) OR elevated plasma IL-10 levels (>20 pg/ml) OR elevated serum or plasma vitamin B12 levels (>1500 ng/L) OR elevated plasma IL-18 levels (>500 pg/ml)
2. Typical immunohistological findings (as reviewed by an experienced pathologist)
3. Autoimmune cytopenias (hemolytic anemia, thrombocytopenia, or neutropenia) AND elevated immunoglobulin G levels (polyclonal hypergammaglobulinemia)
4. Family history of nonmalignant/noninfectious lymphoproliferation with or without autoimmunity

(B) Subtypes of ALPS		
Diagnosis	Definition	Comment
ALPS-FAS	Germline *FAS* mutation	Most common ALPS subgroup, heterozygous mutations leading to haploinsufficiency or negative transdominance, homozygous germline mutations or an additional somatic mutation in *FAS* lead to more severe disease
ALPS-sFAS	Somatic *FAS* mutation	Somatic mutations in FAS, detected in sorted double-negative T cells
ALPS-FASL	Germline *FASL* mutation	Typical ALPS phenotype, SLE-like disease and recurrent bacterial and viral infections have been reported
ALPS-CASP10	Germline *CASP10* mutation	Two pathogenic *CASP10* mutations (I406L and L258F) and two additional (V410l and Y446C) with controversial pathogenicity have been reported so far^135^
ALPS-U	No detected genetic lesion	Patients meet the revised diagnostic criteria, genetic defect is undetermined

A definite diagnosis can be made in the case that both required criteria and at least 1 primary accessory criterion are fulfilled. A probable diagnosis can be made in the presence of both required criteria and at least 1 secondary accessory criterion

Immune dysregulation in ALPS is attributed to defects in lymphocyte apoptosis and especially aberrations in Fas-mediated extrinsic apoptosis.^138,139^ T-cell activation induces the expression of FasL, which binds Fas on nearby cells, including T cells. Fas is a death receptor containing intracellular death domains. The activation of Fas leads to the formation of the death-inducing signaling complex, which recruits and activates caspases 8 and 10, which are initiator caspases of the extrinsic apoptotic pathway. The majority of ALPS patients harbor heterozygous germline mutations in *FAS* (Table 3B).^134^ Incomplete penetrance and variable expressivity of the same *FAS* variants suggest the role of additional genetic modifiers.^140^ Consistent with the latter is the observation of an additional somatic *FAS* mutation accounting for a more severe phenotype in some patients.^141,142^ Germline mutations in the genes encoding FasL (*FASLG*) or caspase 10 (*CASP10*) have been identified in a small subgroup of patients with ALPS.^133,134,143^ The identification of *NRAS* and *KRAS* mutations in patients with ALPS and a propensity for hematopoietic malignancies suggests that in addition to extrinsic apoptosis, defects in the intrinsic apoptotic pathway can cause immune dysregulation.^144,145^

## Type i interferon-mediated immune dysregulation

Type I interferons have numerous effects on the innate and adaptive immune systems.^146^ In particular, they modulate antigen-presenting function; promote inflammation, apoptosis, myeloid cell activation, and B-cell differentiation; and affect the function of several cells, such as microglial and endothelial cells. The activation of type I interferon-mediated responses can contribute to the pathomechanism of autoimmunity. Type I interferonopathies are a group of monogenic disorders caused by abnormal upregulation of type I interferons and typically manifest as vasculopathy, early-onset SLE or myositis.^147,148^ Known type I interferonopathies and their main clinical findings are listed in Table 4. Type I interferonopathies reflect the pathogenic role of constitutive activation of type I interferon-mediated immune responses.^146,147^ Defects in nucleic acid sensing, including the chemical modification or clearance of self-nucleic acids, deregulated activation of nucleic acid sensors and defects in molecules or pathways that regulate type I interferon signaling, can all result in diseases falling under type I interferonopathies. For example, mutations in genes encoding nucleases, such as TREX1 and RNase H2, which cause the prototype type I interferonopathy, Aicardi-Goutières syndrome (AGS), suggest the pathogenic role of the accumulation of self-nucleic acids, which results in systemic inflammation and autoimmunity by activating type I interferon responses.

**Table 4:** Type I inferferonopathies

Disease	Gene	Protein function	Inheritance	Main clinical findings	Reference
**Aicardi-Goutières syndrome (AGS)**	*TREX1*	3′ repair exonuclease (cytosolic DNase)	AR, de novo dominant	Leukoencephalopathy, microcephaly, calcification of basal ganglia, seizures, fever, severe developmental delay, chilblain lesions	^260^
	*RNASEH2A*	Ribonuclease H2, subunit A	AR		
	*RNASEH2B*	Ribonuclease H2, subunit B	AR		
	*RNASEH2C*	Ribonuclease H2, subunit C	AR		
	*ADAR1*	adenosine deaminase, RNA-specific	AR, de novo dominant		
	*IFIH1*	IFN-induced helicase C domain-containing protein 1 (PRR for dsRNA)	AD, de novo dominant		
	*SAMHD1*	SAM domain and HD domain-containing protein 1 (RNase)	AR		
**STING-associated vasculopathy, infantile-onset (SAVI)**	*STING*	Stimulator of interferon genes (induction of type I interferons in response to infection with intracellular pathogens)	AD, de novo dominant	Vasculitis characterized by ulcerating acral lesions, ILD, chilblain lesions, malar rash, arthralgia	^261^
**Familial chilblain lupus (CHBL)**	*TREX1*	3′ repair exonuclease (cytosolic DNase)	AD	Cutaneous lupus erythematosus with chilblain lesions, arthralgia	^262^
	*STING*	Stimulator of interferon genes (induction of type I interferons in response to infection with intracellular pathogens)	AD		
	*SAMHD1*	SAM domain and HD domain-containing protein 1 (RNase)	AD		
**Spondyloenchondrodysplasia (SPENCD)**	*ACP5*	Tartrate-resistant acid phosphatase, type 5 (dephosphorylation of osteopontin)	AR	Spondylometaphyseal dysplasia, basal ganglia calcification, variable neurological impairment, arthritis, thrombocytopenia, variable immunodeficiency resulting in recurrent pneumonias, severe VZV infection/reactivation, mucocutaneous abscesses (in a minority of patients)	^263^
**Chronic atypical neutrophilic dermatosis with lipodystrophy and elevated temperature (CANDLE)**	*PSMA3*	Proteasome subunit α7 (immunoproteasome subunit)	AR	Eczema, panniculitis, lipodystrophy, fever, calcification of basal ganglia	^264^
	*PSMB4*	Proteasome subunit β7 (immunoproteasome subunit)	AR		
	*PSMB8*	Proteasome subunit β5i (immunoproteasome subunit)	AR		
	*PSMB9*	Proteasome subunit β1i (immunoproteasome subunit)	AR		
	*POMP*	Proteasome maturation protein (immunoproteasome formation)	AR		
**Renal vasculopathy with cerebral leukodystrophy (RVCL)**	*TREX1*	3’ repair exonuclease (cytosolic DNase)	AD	Retinopathy, leukodystrophy, cerebrovascular disease, glomerulopathy	^265^
**Singleton-Merten syndrome (SGMRT)**	*IFIH1*	IFN-induced helicase C domain-containing protein 1 (PRR for dsRNA)	AD	Progressive vasculopathy with the calcification of large vessels, dental and skeletal abnormalities, osteoporosis, photosensitivity, psoriasis	^266,267^
	*RIG1*	Retinoic acid-inducible gene 1 (PRR for dsRNA)	AD		
**ISG15 deficiency**	*ISG15*	Interferon-stimulated gene 15 (protein ISGylation, IFNγ induction)	AR	Calcification of basal ganglia, necrotizing skin lesions, MSMD	^268^
**USP18 deficiency**	*USP18*	Ubiquitin-specific protease 18 (de- ISGylation)	AR	Microcephaly, cerebral calcification, thrombocytopenia	^269^
**X-linked reticulate pigmentary disorder (XLPDR)**	*POLA1*	DNA polymerase α (DNA replication)	XR	Hyperpigmented skin lesions, enteropathy, hypogammaglobulinemia-associated respiratory tract infections and invasive fungal infections (in a minority of patients)	^270^ Formularbeginn Formularende
**DNase II deficiency**	*DNASE2*	Deoxyribonuclease II (lysosomal endonuclease)	AR	Hepatosplenomegaly, cutaneous vasculitis lesions, recurrent fever, endocrinopathy, anemia, thrombocytopenia, glomerulonephritis, hypogammaglobulinemia necessitating immunoglobulin replacement (in a minority of patients)	^271^

*AD* autosomal dominant, *AR* autosomal recessive, *HD* histidine-aspartic, *ILD* interstitial lung disease, *MSMD* Mendelian susceptibility to mycobacterial disease, *PRR* pattern recognition receptor, *SAM* sterile alpha motif, *VZV* varicella zoster virus, *XR* X-linked recessive

### STAT1 gain of function

Signal transducer and activator of transcription 1 (STAT1) is involved in both type I (IFNα and IFNβ) and type II (IFNγ) interferon receptor signaling.^149^ The activation of the IFNγ receptor results in phosphorylation and STAT1, which forms a homodimer that translocates in the nucleus, where it induces IFNγ-regulated genes. Stimulation with type I interferons in addition to STAT1 homodimers results in the formation of a STAT1-STAT2 and p48 (ISGF3G) heterotrimer that transactivates type I interferon-regulated genes, whose collective induction is referred to as the type I interferon signature. Heterozygous gain-of-function mutations in *STAT1* result in immunodeficiency, typically manifesting as chronic mucocutaneous candidiasis (CMC).^150^ However, a significant proportion of patients (37%) display autoimmune manifestations, including endocrinopathy, autoimmune cytopenia, and SLE. *STAT1* gain-of-function mutations do not affect the expression of FOXP3 or CTLA-4 or the development of Tregs.^151^ Despite the phenotypic overlap between APECED and immunodeficiency due to STAT1 gain of function, there is no evidence of a pathomechanistic connection between these two monogenic disorders. Considering the phenotypic similarities of interferonopathies such as SLE and the fact that treatment with interferon α results in endocrinopathy (such as thyroiditis), enhanced cytokine and interferon responses provide a plausible pathomechanism accounting for autoimmunity in the context of STAT1 gain of function.^152,153^ The recently identified enhanced expression of interferon signature genes in patients with *STAT1* gain-of-function mutations^154^ is in accordance with the aforementioned hypothesis. Although, so far, there is no clear genotype-phenotype correlation, considering the varying gain-of-function effect of *STAT1* variants, it would be interesting to evaluate whether autoimmunity associates with *STAT1* variants resulting in stronger interferon signaling.

### Deficiency of ADA2

Patients with a deficiency in adenosine deaminase 2 (ADA2) due to biallelic loss-of-function mutations in *CECR1* display varying immunodeficiency mainly associated with hypogammaglobulinemia but also early-onset polyarteritis nodosa.^155,156^ The severity of polyarteritis nodosa in those patients ranges from cutaneous vasculitis to organ involvement, including early-onset and/or recurrent strokes. ADA2 is mainly expressed by myeloid cells and catalyzes the conversion of adenosine and 2′-deoxyadenosine to inosine and deoxyinosine, respectively. In contrast to adenosine deaminase 1 (ADA1), whose deficiency results in SCID, ADA2 has a considerably lower affinity for its substrates and is not acting intracellularly but is rather secreted. Phenotypic similarities between ADA2 deficiency (DADA2) and type I interferonopathies, such as AGS, suggest a shared pathogenic background.^157^ In accordance with this is the fact that DADA2 patients display a type I interferon signature correlating with disease activity, as shown in transcriptome analysis of their peripheral blood cells.^157,158^ The identification of DADA2 as a monogenic form of polyarteritis nodosa suggests that defects in adenosine catabolism result in inflammation by inducing the production of type I interferons.

## B-cell-intrinsic defects

Autoreactive B cells are subject to the mechanisms of central tolerance, which are deployed within the bone marrow and include clonal deletion and receptor editing.^1,159^ However, similar to T cells, the central tolerance of B cells is incomplete, and as a consequence, a considerable proportion of B cells escaping the bone marrow are autoreactive. The frequency of autoreactive B cells decreases along the course of B-cell maturation, falling from the staggering level of more than 55% within the population of early immature B cells to ~20% within mature B cells.^1,160,161^ The control of this sizable proportion of autoreactive mature B cells can be achieved through anergy, clonal ignorance, clonal deletion, and receptor revision. Somatic hypermutation in the context of the germinal center reaction can convert previously nonautoreactive cells into autoreactive cells. Germinal center checkpoints are employed in this case to prevent the breakdown of tolerance and are largely the mechanism inducing T-cell tolerance. T cells are gatekeepers of B-cell tolerance, and mechanisms of T-cell tolerance are relevant for the induction of B-cell tolerance.

### Autosomal-recessive AID deficiency

Activation-induced cytidine deaminase (AID) catalyzes the deamination of cytosine into uracil, creating DNA mutations, and AID is involved in immunoglobulin class-switch recombination (CSR) and somatic hypermutation.^162^ Biallelic mutations in *AICDA* result in AID deficiency, which is the most common B-cell-intrinsic CSR defect.^163,164^ Autosomal-recessive AID deficiency causes hyper-IgM syndrome with low IgG and IgA levels. Apart from recurrent bacterial infections, reflecting antibody failure, the majority of patients display lymphadenopathy, and ~30% develop autoimmunity, presenting as autoimmune cytopenia, SLE, arthritis, autoimmune hepatitis, and Crohn’s disease. Autoimmunity in AID deficiency is a B-cell-intrinsic defect.^165^ Both defects in central and peripheral tolerance have been described, though the exact mechanism accounting for B-cell autoreactivity is not clearly understood. The resistance of B cells to apoptosis and the overexpression of BAFF could result in the skipping of the checkpoints of B-cell tolerance in these patients.^166^ An additional pathogenic correlate of autoimmunity in AID deficiency is the reduction in Treg counts, which may be explained through a T-cell-intrinsic mechanism, as T cells have been reported to transiently express AID.^167^ Further, the presentation of autoantigens by autoreactive B cells has been suggested as an alternative mechanism promoting the activation of autoreactive T cells.^168^

### PKCδ deficiency

Protein kinase C δ (PKCδ) is a lymphocyte and primarily B-cell signaling mediator.^169^ PKCδ is phosphorylated in response to the activation of the BCR B-cell activating factor (BAFF) or in response to cytokines such as IL-4 and interferons. PKCδ-mediated signaling in B cells regulates their survival, proliferation, and apoptosis, including the proapoptotic pathway resulting in negative selection. Biallelic loss-of-function mutations in *PRKCD*, the gene encoding PKCδ, either result in PKCδ deficiency or affect its phosphorylation; in addition, these mutations cause immunodeficiency with variable immunoglobulin values and consistently reduced counts of class-switched memory B cells, characterized by immune dysregulation.^170–173^ Six patients from four unrelated families have been reported to develop disease as a consequence of biallelic *PRKCD* mutations. All of these patients developed autoimmunity before the age of 10 years, commonly manifesting as SLE or SLE-like disease, including photosensitivity and nephritis, arthritis, and antiphospholipid syndrome. The critical role of PKCδ in B cell homeostasis and tolerance, especially in negative selection in germinal centers, has been identified in PKCδ-deficient mice, which – similar to patients with PKCδ deficiency—display lymphoproliferation and systemic autoimmunity.^174^

## Uncontrolled infections and autoimmunity: The example of failed control of ebv and mycobacteria

Pathogens of all kinds can cause tissue damage and elicit both innate and adaptive immune responses that not only eliminate them but also may result in the breakdown of tolerance.^175–177^ The mechanisms by which infections can trigger or accelerate autoimmunity have been extensively reviewed elsewhere.^178–180^ Briefly, mechanisms accounting for infection-triggered autoimmunity include the release of self-antigens, enhanced antigen presentation, bystander activation, superantigen-induced immune activation, and molecular mimicry. Mechanistic insight into the pathogenesis of autoimmunity has been gained through animal models of autoimmune disease, which also provides a platform for the evaluation of the role of particular viruses and bacteria in the initiation and maintenance of autoimmunity.^181^ Epidemiological studies have associated several autoimmune diseases, such as T1D, RA, SS, and SLE, with viral or bacterial infections.^182–186^ Further, pathogen-derived antigens have been identified in tissues or cells of patients with autoimmune diseases,^182,185^ and sequence similarities between pathogen-derived epitopes and self-antigen-derived epitopes that could elicit cross-reacting immune responses have been identified in numerous autoimmune diseases.^187–190^ Given the evidence suggesting the role of infections in precipitating autoimmunity, it is tempting to speculate that, at least in some IEI, susceptibility to infections, and especially chronic or persistent infections, may precipitate autoimmunity. As this is an extremely broad topic that should be addressed with a pathogen-specific approach, we will focus here on EBV, whose failed control is a hallmark of T-cell immunodeficiency and atypical mycobacterial infections, which are associated with Mendelian susceptibility to mycobacterial disease (MSMD).

Several lines of evidence connect EBV with autoimmunity. Epidemiological studies have associated EBV with autoimmune diseases such as RA, SLE and multiple sclerosis (MS).^178,190^ EBV-induced infectious mononucleosis has been suggested to induce the production of various autoantibodies, such as those against DNA, ribonucleoproteins, and erythrocytes.^182,191,192^ Further, EBV-derived proteins and nucleic acids have been more commonly identified in patients with SLE. In SLE patients, it has been shown that EBV-specific CD8^+^ T cells are diminished and less cytotoxic.^193,194^ Decreased CD8^+^ T-cell activity against EBV-infected B cells has been suggested to account for the accumulation of EBV-infected B cells in MS.^195^ RA patients displayed decreased percentages of IFNγ-producing EBV-specific CD8^+^ T cells.^196^ The abovementioned findings link defective control of EBV with autoimmunity. Several IEI primarily compromise the handling of EBV.^197^ For example, mutations in *ITK, CD27, MAGT1, and CORO1A* result in failed control of EBV infection and EBV-driven lymphoproliferation.^198^ Autoimmunity is neither a cardinal nor an early-onset feature of most of those disorders and has been reported in a minority of patients with ITK deficiency or X-linked immunodeficiency with magnesium defect, EBV infection and neoplasia (XMEN) syndrome who more commonly developed autoimmune cytopenia.^199,200^ Considering the plausible epidemiological and biological evidence of the role of EBV in autoimmunity, it can be speculated that EBV-driven mechanisms in the context of failed EBV control can at least partially induce the breakdown of tolerance in PIDs that selectively affect immune responses against EBV.

Mycobacterial infections, especially with nontuberculous mycobacteria, can induce autoantibody responses, such as those found in SLE, RA, and systemic vasculitis, and *M. tuberculosis*-derived antigens, such as heat shock protein 60 (HSP60) and HSP65, have been identified in the sera of patients with SLE.^201–203^ Nontuberculous mycobacterial infections have a relatively high prevalence among SLE patients.^204^ Epidemiological studies revealed a significant association of SjS with a history of previous nontuberculous mycobacterial infection.^205^ In addition to molecular mimicry, persistent antigenic stimulation in the context of mycobacterial infection could activate innate immune sensors and lower the activation threshold of autoreactive T cells.^202^ Common IEI resulting in MSMD,^206^ such as interleukin 12 receptor beta 1 (IL12Rβ1) or interferon γ receptor 1 (IFNγR1) deficiency, has only rarely been linked to autoimmunity, which manifests as SjS or SLE-like disease.^207–210^ The relatively low proportion of patients with monogenic defects in the IL-12*/*IFNγ axis and autoimmunity (3.4% among patients with IL12Rβ1 deficiency)^207^ suggests an additional genetic predisposition to autoimmunity other than the MSMD-causing genetic variation. Given the evidence suggesting that mycobacteria can precipitate autoimmunity, persistent mycobacterial infection may at least in part account for the breakdown of tolerance in these patients.

## Multifactorial immune dysregulation: the example of IEI due to defects in the canonical NF-κB pathway

The activation of NF-κB transcription factors, especially those of the canonical pathway, plays a central role in the immune system and is a ubiquitous target of immune signaling^211^; therefore, it is discussed separately in this review. Diverse immune receptors, including antigen receptors, Toll-like receptors (TLRs), members of the tumor necrosis factor receptors (TNFRs) and the interleukin 1 receptor (IL-1R), activate the canonical NF-κB pathway, which converges in the nuclear translocation of an NF-κB dimer, which consists of c-Rel, RelA/p65 and/or p50.^211^ Heterozygous loss-of-function mutations in *NFKB1*, the gene encoding the precursor of p50, i.e., NF-κB1/p105, are the most common monogenic cause of CVID in Europeans, and, in addition to immunodeficiency, they are commonly associated with autoimmunity.^212^ More than half of patients with immunodeficiency due to damaging monoallelic *NFKB1* mutations display autoimmune manifestations such as autoimmune cytopenias, arthritis, enteropathy, and vasculitis, and the frequency of autoimmunity increases with age.^213,214^ Heterozygous gain-of-function mutations in *NFKBIA*, the gene encoding the “nuclear factor of kappa light polypeptide gene enhancer in B cells inhibitor alpha” (IκBα), block IκBα degradation and therefore NF-κB1 release from its complex with IκBα and nuclear translocation, having a loss-of-function effect on the canonical NF-κB pathway.^215^ Approximately half of all 19 reported patients with PID due to *NFKBIA* mutations displayed autoimmune or autoinflammatory disease manifesting as SLE, RA, IBD, or hepatopathy.^215–219^

Despite mouse studies suggesting the involvement of the canonical NF-κB pathway in the development and function of Tregs,^220,221^ both Treg counts and in vitro suppressive function appear normal in tested patients with immunodeficiency due to monoallelic *NFKB1* mutations.^222^ However, a recent study revealed reduced expression of ICOS in Tregs from all 5 tested patients with NFKB1 haploinsufficiency, which is consistent with the reduced effector function of Tregs.^223^ Those patients displayed reduced Treg counts, deviating from the rest of patients with monoallelic *NFKB1* mutations,^222^ so it is unclear whether all pathogenic *NFKB1* mutations consistently impair Treg counts or function and whether this is associated with autoimmune disease. Dysregulated stimulation-induced cytokine secretion and especially increased production of TNF can result in autoimmunity.^224^ Increased production of TNF has been described in patients with heterozygous *NFKB1* mutations and may be an additional or alternative explanation for autoimmune or autoinflammatory disease in the context of immunodeficiency due to heterozygous *NFKB1* mutations. Myeloid cell hyperactivation and the oversecretion of IL-1b have been reported to be the consequence of a recently identified gain-of-function *NFKBIA* mutation in a patient with autoinflammatory hepatopathy.^219^ Another mutation in *NFKBIA*, replacing Ser32, did not cause the oversecretion of IL-1β.^225^ However, the patient’s leukocytes displayed reduced LPS-induced production of all other tested proinflammatory cytokines (IL-6 and TNFα), except IL-1β, supporting the idea of a cytokine imbalance favoring IL-1β production as the cause of inflammatory disease in these patients.

The activation of the canonical NF-κB pathway, especially after triggering TCR or BCR, is controlled by the assembly of a protein complex consisting of CARD11, BCL-10, and MALT1, the so-called CBM signalosome.^226^ Germline mutations in the genes encoding the components of the CBM signalosome result in a broad spectrum of immunological phenotypes, including combined immunodeficiency, atopy, and autoimmunity, which is more clearly linked with Treg dysfunction. Germline gain-of-function mutations in *CARD11* underlie B-cell expansion with NF-κB and T-cell anergy (BENTA) disease, which is a PID with recurrent respiratory tract and viral infections such as molluscum contagiosum and EBV viremia.^227^ BENTA patients also display autoimmune lymphoproliferative syndrome (ALPS)-like lymphoproliferation and SLE-like autoimmunity, including autoimmune cytopenias. Most BENTA-causing mutations are located in the coiled-coil or the LATCH domain of CARD11, interrupting the autoinhibitory interaction with the linker domain, resulting in CARD11 aggregation, the recruitment of BCL-10 and MALT1 and constitutive NF-κB activation in B cells and T cells.^226,228^ Interestingly, dominant-negative (DN) CARD11 mutations disrupting NF-κB activation have also been associated with autoimmunity.^229^ In addition to severe atopic disease, ~20% of patients with DN *CARD11* mutations display autoimmunity manifesting as alopecia, idiopathic thrombocytopenic purpura, bullous-pemphigoid, or even IPEX-like disease. Although severe opportunistic infections (particularly *Pneumocystis* pneumonia) dominate the phenotype of combined immunodeficiency due to biallelic loss-of-function mutations in *CARD11*, Omenn syndrome and IBD have been reported as manifestations of PID due to full CARD11 deficiency, which results in compromised activation of the canonical NF-κB pathway and an absence of Tregs.^226,230^ MALT1 deficiency, as a consequence of germline biallelic loss-of-function mutations in *MALT1*, causes CID, including IPEX-syndrome-like immune dysregulation, which is associated with substantially reduced Treg counts.^231^ The IPEX-syndrome-like autoimmunity of MALT1-deficient patients is in accordance with the phenotype of mice harboring a point mutation that selectively inactivates the paracaspase activity of MALT1, which also display reduced Treg counts and die of multiorgan autoimmunity.^232^ BCL10 deficiency, similar to MALT1 deficiency, causes an IPEX-syndrome-like phenotype associated with profound Treg deficiency.^233^

## Concluding remarks

Autoimmunity is an integral part of the clinical spectrum of PIDs or IEI. In this review, we have presented the cellular and molecular mechanisms accounting for the breakdown of tolerance to self-antigens in selected IEI (Figs. 1 and 2).

The age of onset and the type of autoimmunity depend largely on the genetic defect underlying an IEI and its impact on both immunity and tolerance. Major defects in thymic negative selection commonly cause early-onset polyendocrinopathy and cutaneous autoimmunity, whereas major defects in Treg-induced peripheral tolerance typically manifest with early-onset enteropathy, endocrinopathy, and eczema. Several genetic defects, especially those affecting antigen receptor signaling or nodal signaling molecules, can simultaneously impair more than one layer of tolerance. On the other hand, unresolved infections may directly affect tolerogenic lymphocyte functions or exert a persisting adjuvant effect on immune cells, precipitating the breakdown of tolerance. The latter may be relevant for monogenic immune defects that predominantly compromise immunity and are associated with a lower prevalence and a later onset of autoimmunity.

**Fig. 3:**
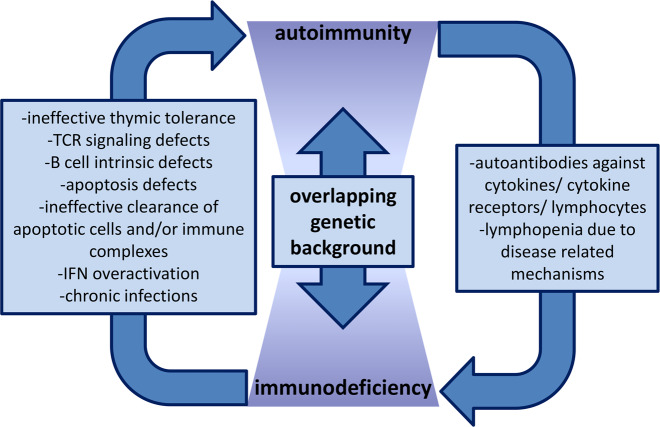
The interconnected pathogenic pathways of autoimmunity and primary immunodeficiency. Autoimmune disorders may result in immunodeficiency through the production of autoantibodies or through disease-intrinsic mechanisms, whereas the immune defects underlying immunodeficiency can affect the induction or the maintenance of immune tolerance and cause autoimmunity

Autoimmunity can be the prominent or the first manifestation of an IEI.^10^ In the case of the latter scenario, the diagnosis of primary immunodeficiency may be missed, as patients may receive glucocorticoids or other immunomodulatory agents inducing secondary immunodeficiency.^234^ The increasing number of genes linked to IEI facilitates an understanding of the immunopathogenesis of autoimmune disease, affecting therapeutic decision-making or even allowing the development of individualized therapies. The emerging identification of genes both involved in IEI and conferring susceptibility to systemic autoimmune diseases, as well as the increasing identification of autoantibodies and other intrinsic autoimmune disease mechanisms compromising immunity, strongly suggest the interconnectedness of the pathogenic pathways of autoimmunity and primary immunodeficiency (Fig. 3).^235–237^ Therefore, the identification of the mechanisms breaking immune tolerance in IEI may aid in the understanding of the pathophysiology of systemic autoimmune diseases and contribute to the development of pathophysiology-oriented therapeutics.

## References

[1] Theofilopoulos AN, Kono DH, Baccala R (2017). The multiple pathways to autoimmunity. Nat. Immunol..

[2] Imgenberg-Kreuz, J., Rasmussen, A., Sivils, K. & Nordmark, G. Genetics and epigenetics in primary Sjögren’s syndrome. *Rheumatology (Oxford)*. 10.1093/rheumatology/key330. (2019). 10.1093/rheumatology/key330 PMC8121440 30770922

[3] Okada Y, Eyre S, Suzuki A, Kochi Y, Yamamoto K (2019). Genetics of rheumatoid arthritis: 2018 status. Ann. Rheum. Dis..

[4] Demirkaya E, Sahin S, Romano M, Zhou Q, Aksentijevich I (2020). New horizons in the genetic etiology of systemic lupus erythematosus and lupus-like disease: monogenic lupus and beyond. J. Clin. Med..

[5] Ishigaki K, Kochi Y, Yamamoto K (2018). Genetics of human autoimmunity: From genetic information to functional insights. Clin. Immunol..

[6] Bousfiha A (2020). Human inborn errors of immunity: 2019 update of the IUIS phenotypical classification. J. Clin. Immunol..

[7] Gruber C, Bogunovic D (2020). Incomplete penetrance in primary immunodeficiency: a skeleton in the closet. Hum. Genet..

[8] Delmonte, O. M., Castagnoli, R., Calzoni, E. & Notarangelo, L. D. Inborn errors of immunity with immune dysregulation: from bench to bedside. *Front. Pediatr.* **7**, 353 (2019). 10.3389/fped.2019.00353 PMC6718615 31508401

[9] Grimbacher B, Warnatz K, Yong PFK, Korganow AS, Peter HH (2016). The crossroads of autoimmunity and immunodeficiency: Lessons from polygenic traits and monogenic defects. J. Allergy Clin. Immunol..

[10] Sogkas, G. et al. High frequency of variants in genes associated with primary immunodeficiencies in patients with rheumatic diseases with secondary hypogammaglobulinaemia. *Ann. Rheum. Dis*. 10.1136/annrheumdis-2020-218280 (2020). 10.1136/annrheumdis-2020-218280 33046446

[11] Warnatz K, Voll RE (2012). Pathogenesis of autoimmunity in common variable immunodeficiency. Front. Immunol..

[12] Grammatikos AP, Tsokos GC (2012). Immunodeficiency and autoimmunity: lessons from systemic lupus erythematosus. Trends Mol. Med..

[13] Ku CL, Chi CY, von Bernuth H, Doffinger R (2020). Autoantibodies against cytokines: phenocopies of primary immunodeficiencies?. Hum. Genet..

[14] Merkel PA, Lebo T, Knight V (2019). Functional analysis of anti-cytokine autoantibodies using flow cytometry. Front. Immunol..

[15] Baerlecken N (2009). Recurrent, multifocal Mycobacterium avium-intercellulare infection in a patient with interferon-gamma autoantibody. Clin. Infect. Dis..

[16] Asakura T (2017). Disseminated mycobacterium genavense infection in patient with adult-onset immunodeficiency. Emerg. Infect. Dis..

[17] Rosen LB (2013). Anti-GM-CSF autoantibodies in patients with cryptococcal meningitis. J. Immunol..

[18] Crum-Cianflone NF, Lam PV, Ross-Walker S, Rosen LB, Holland SM (2017). Autoantibodies to granulocyte-macrophage colony-stimulating factor associated with severe and unusual manifestations of *Cryptococcus gattii* infections. Open Forum Infect. Dis..

[19] Trapnell BC (2019). Pulmonary alveolar proteinosis. Nat. Rev. Dis. Prim..

[20] Bastard P (2020). Auto-antibodies against type I IFNs in patients with life-threatening COVID-19. Science.

[21] Bluestone JA (2011). Mechanisms of tolerance. Immunol. Rev..

[22] Lourenço EV, La Cava A (2011). Natural regulatory T cells in autoimmunity. Autoimmunity.

[23] Kanamori M, Nakatsukasa H, Okada M, Lu Q, Yoshimura A (2016). Induced regulatory T Cells: their development, stability, and applications. Trends Immunol..

[24] Constantine GM, Lionakis MS (2019). Lessons from primary immunodeficiencies: autoimmune regulator and autoimmune polyendocrinopathy-candidiasis-ectodermal dystrophy. Immunol. Rev..

[25] Perniola R (2018). Twenty years of AIRE. Front. Immunol..

[26] Puel A (2010). Autoantibodies against IL-17A, IL-17F, and IL-22 in patients with chronic mucocutaneous candidiasis and autoimmune polyendocrine syndrome type I. J. Exp. Med..

[27] Kisand K (2010). Chronic mucocutaneous candidiasis in APECED or thymoma patients correlates with autoimmunity to Th17-associated cytokines. J. Exp. Med..

[28] Giardino G (2019). Clinical and immunological features in a cohort of patients with partial DiGeorge syndrome followed at a single center. Blood.

[29] Davies EG (2013). Immunodeficiency in DiGeorge syndrome and options for treating cases with complete athymia. Front. Immunol..

[30] Sullivan KE, McDonald-McGinn D, Zackai EH (2002). CD4(+) CD25(+) T-cell production in healthy humans and in patients with thymic hypoplasia. Clin. Diagn. Lab. Immunol..

[31] Gennery AR (2002). Antibody deficiency and autoimmunity in 22q11.2 deletion syndrome. Arch. Dis. Child..

[32] Klemann C (2019). Clinical and immunological phenotype of patients with primary immunodeficiency due to damaging mutations in *NFKB2*. Front. Immunol..

[33] Zhu M (2006). NF-kappaB2 is required for the establishment of central tolerance through an Aire-dependent pathway. J. Clin. Invest..

[34] Zhang B (2006). NF-kappaB2 is required for the control of autoimmunity by regulating the development of medullary thymic epithelial cells. J. Biol. Chem..

[35] Tucker E (2007). A novel mutation in the Nfkb2 gene generates an NF-kappa B2 “super repressor”. J. Immunol..

[36] Maccari ME (2017). Severe Toxoplasma gondii infection in a member of a NFKB2-deficient family with T and B cell dysfunction. Clin. Immunol..

[37] Lee CE (2014). Autosomal-dominant B-cell deficiency with alopecia due to a mutation in NFKB2 that results in nonprocessable p100. Blood.

[38] Grinberg-Bleyer Y (2018). The alternative NF-κB pathway in regulatory T cell homeostasis and suppressive function. J. Immunol..

[39] Villa A, Notarangelo LD, Roifman CM (2008). Omenn syndrome: inflammation in leaky severe combined immunodeficiency. J. Allergy Clin. Immunol..

[40] Cavadini P (2005). AIRE deficiency in thymus of 2 patients with Omenn syndrome. J. Clin. Invest..

[41] Cassani B (2010). Defect of regulatory T cells in patients with Omenn syndrome. J. Allergy Clin. Immunol..

[42] Mouchess ML, Anderson M (2014). Central tolerance induction. Curr. Top. Microbiol. Immunol..

[43] Malhotra D (2016). Tolerance is established in polyclonal CD4(+) T cells by distinct mechanisms, according to self-peptide expression patterns. Nat. Immunol..

[44] Tivol EA (1995). Loss of CTLA-4 leads to massive lymphoproliferation and fatal multiorgan tissue destruction, revealing a critical negative regulatory role of CTLA-4. Immunity.

[45] Zhang X, Olsen N, Zheng SG (2020). The progress and prospect of regulatory T cells in autoimmune diseases. J. Autoimmun..

[46] Wildin RS (2001). X-linked neonatal diabetes mellitus, enteropathy and endocrinopathy syndrome is the human equivalent of mouse scurfy. Nat. Genet..

[47] Cepika AM (2018). Tregopathies: monogenic diseases resulting in regulatory T-cell deficiency. J. Allergy Clin. Immunol..

[48] Jamee M (2020). Clinical, immunological, and genetic features in patients with Immune Dysregulation, Polyendocrinopathy, Enteropathy, X-linked (IPEX) and IPEX-like Syndrome. J. Allergy Clin. Immunol. Pr..

[49] Park JH (2020). Immune dysregulation, polyendocrinopathy, enteropathy, X-linked (IPEX) syndrome: A systematic review. Autoimmun. Rev..

[50] Verma N, Burns SO, Walker LSK, Sansom DM (2017). Immune deficiency and autoimmunity in patients with CTLA-4 (CD152) mutations. Clin. Exp. Immunol..

[51] Yokosuka T (2010). Spatiotemporal basis of CTLA-4 costimulatory molecule-mediated negative regulation of T cell activation. Immunity.

[52] Hogquist KA, Jameson SC (2014). The self-obsession of T cells: how TCR signaling thresholds affect fate ‘decisions’ and effector function. Nat. Immunol..

[53] Waterhouse P (1995). Lymphoproliferative disorders with early lethality in mice deficient in Ctla-4. Science.

[54] Mitsuiki N, Schwab C, Grimbacher B (2019). What did we learn from CTLA-4 insufficiency on the human immune system?. Immunol. Rev..

[55] Kuehn HS (2014). Immune dysregulation in human subjects with heterozygous germline mutations in CTLA4. Science.

[56] Schwab C (2018). Phenotype, penetrance, and treatment of 133 cytotoxic T-lymphocyte antigen 4-insufficient subjects. J. Allergy Clin. Immunol..

[57] Lo B (2015). Patients with LRBA deficiency show CTLA4 loss and immune dysregulation responsive to abatacept therapy. Science.

[58] Tesch VK (2020). Long-term outcome of LRBA deficiency in 76 patients after various treatment modalities as evaluated by the immune deficiency and dysregulation activity (IDDA) score. J. Allergy Clin. Immunol..

[59] Charbonnier LM (2015). Regulatory T-cell deficiency and immune dysregulation, polyendocrinopathy, enteropathy, X-linked-like disorder caused by loss-of-function mutations in LRBA. J. Allergy Clin. Immunol..

[60] Gámez-Díaz L (2016). The extended phenotype of LPS-responsive beige-like anchor protein (LRBA) deficiency. J. Allergy Clin. Immunol..

[61] Kiykim A (2019). Abatacept as a long-term targeted therapy for LRBA deficiency. J. Allergy Clin. Immunol. Pr..

[62] Fos C (2014). Association of the EF-hand and PH domains of the guanine nucleotide exchange factor SLAT with IP_3_ receptor 1 promotes Ca²^+^ signaling in T cells. Sci. Signal..

[63] Fanzo JC (2016). Loss of IRF-4-binding protein leads to the spontaneous development of systemic autoimmunity. J. Clin. Invest..

[64] Canonigo-Balancio AJ, Fos C, Prod’homme T, Bécart S, Altman A (2009). SLAT/Def6 plays a critical role in the development of Th17 cell-mediated experimental autoimmune encephalomyelitis. J. Immunol..

[65] Serwas NK (2019). Human DEF6 deficiency underlies an immunodeficiency syndrome with systemic autoimmunity and aberrant CTLA-4 homeostasis. Nat. Commun..

[66] Vignoli M (2019). CD25 deficiency: a new conformational mutation prevents the receptor expression on cell surface. Clin. Immunol..

[67] Goudy K (2013). Human IL2RA null mutation mediates immunodeficiency with lymphoproliferation and autoimmunity. Clin. Immunol..

[68] Bezrodnik L, Caldirola MS, Seminario AG, Moreira I, Gaillard MI (2014). Follicular bronchiolitis as phenotype associated with CD25 deficiency. Clin. Exp. Immunol..

[69] Roth TL (2018). Reprogramming human T cell function and specificity with non-viral genome targeting. Nature.

[70] Sharma R (2007). A regulatory T cell-dependent novel function of CD25 (IL-2Ralpha) controlling memory CD8(+) T cell homeostasis. J. Immunol..

[71] Afzali B (2017). BACH2 immunodeficiency illustrates an association between super-enhancers and haploinsufficiency. Nat. Immunol..

[72] Roychoudhuri R (2013). BACH2 represses effector programs to stabilize T(reg)-mediated immune homeostasis. Nature.

[73] Sidwell T (2020). Attenuation of TCR-induced transcription by Bach2 controls regulatory T cell differentiation and homeostasis. Nat. Commun..

[74] Bernasconi A (2006). Characterization of immunodeficiency in a patient with growth hormone insensitivity secondary to a novel STAT5b gene mutation. Pediatrics.

[75] Cheng G, Yu A, Malek TR (2011). T-cell tolerance and the multi-functional role of IL-2R signaling in T-regulatory cells. Immunol. Rev..

[76] Cohen AC (2006). Cutting edge: decreased accumulation and regulatory function of CD4+ CD25(high) T cells in human STAT5b deficiency. J. Immunol..

[77] Acres MJ (2019). Signal transducer and activator of transcription 5B deficiency due to a novel missense mutation in the coiled-coil domain. J. Allergy Clin. Immunol..

[78] Gharibi T (2020). Targeting STAT3 in cancer and autoimmune diseases. Eur. J. Pharm..

[79] Zhang Q, Boisson B, Béziat V, Puel A, Casanova JL (2018). Human hyper-IgE syndrome: singular or plural?. Mamm. Genome.

[80] Sogkas G (2020). Dupilumab to treat severe atopic dermatitis in autosomal dominant hyper-IgE syndrome. Clin. Immunol..

[81] Flanagan SE (2014). Activating germline mutations in STAT3 cause early-onset multi-organ autoimmune disease. Nat. Genet..

[82] Jägle S (2020). Distinct molecular response patterns of activating STAT3 mutations associate with penetrance of lymphoproliferation and autoimmunity. Clin. Immunol..

[83] Nabhani S (2017). STAT3 gain-of-function mutations associated with autoimmune lymphoproliferative syndrome like disease deregulate lymphocyte apoptosis and can be targeted by BH3 mimetic compounds. Clin. Immunol..

[84] Khoury T (2017). Tocilizumab promotes regulatory T-cell alleviation in STAT3 gain-of-function-associated multi-organ autoimmune syndrome. Clin. Ther..

[85] Passerini L (2008). STAT5-signaling cytokines regulate the expression of FOXP3 in CD4+CD25+ regulatory T cells and CD4+CD25- effector T cells. Int. Immunol..

[86] Pillemer BB, Xu H, Oriss TB, Qi Z, Ray A (2007). Deficient SOCS3 expression in CD4+CD25+FoxP3+ regulatory T cells and SOCS3-mediated suppression of Treg function. Eur. J. Immunol..

[87] Biggs CM, Keles S, Chatila TA (2017). DOCK8 deficiency: Insights into pathophysiology, clinical features and management. Clin. Immunol..

[88] Alroqi FJ (2017). DOCK8 deficiency presenting as an IPEX-like disorder. J. Clin. Immunol..

[89] Janssen E (2014). Dedicator of cytokinesis 8-deficient patients have a breakdown in peripheral B-cell tolerance and defective regulatory T cells. J. Allergy Clin. Immunol..

[90] Huber M, Lohoff M (2014). IRF4 at the crossroads of effector T-cell fate decision. Eur. J. Immunol..

[91] Haljasorg U (2017). Irf4 expression in thymic epithelium is critical for thymic regulatory T cell homeostasis. J. Immunol..

[92] Mittrücker HW (1997). Requirement for the transcription factor LSIRF/IRF4 for mature B and T lymphocyte function. Science.

[93] Bravo García-Morato M (2018). New human combined immunodeficiency caused by interferon regulatory factor 4 (IRF4) deficiency inherited by uniparental isodisomy. J. Allergy Clin. Immunol..

[94] Sogkas G, Adriawan IR, Dubrowinskaja N, Atschekzei F, Schmidt RE (2020). Homeostatic and pathogenic roles of PI3Kδ in the human immune system. Adv. Immunol..

[95] Sogkas G (2018). Primary immunodeficiency disorder caused by phosphoinositide 3-kinase δ deficiency. J. Allergy Clin. Immunol..

[96] Conley ME (2012). Agammaglobulinemia and absent B lineage cells in a patient lacking the p85α subunit of PI3K. J. Exp. Med..

[97] Hanna BS (2019). PI3Kδ inhibition modulates regulatory and effector T-cell differentiation and function in chronic lymphocytic leukemia. Leukemia.

[98] Sauer S (2008). T cell receptor signaling controls Foxp3 expression via PI3K, Akt, and mTOR. Proc. Natl Acad. Sci. USA.

[99] Patton DT (2006). Cutting edge: the phosphoinositide 3-kinase p110 delta is critical for the function of CD4+CD25+Foxp3+ regulatory T cells. J. Immunol..

[100] Chellappa S (2019). The PI3K p110δ Isoform Inhibitor Idelalisib Preferentially Inhibits Human Regulatory T Cell Function. J. Immunol..

[101] Sharman JP (2019). Final results of a randomized, phase III study of rituximab with or without idelalisib followed by open-label idelalisib in patients with relapsed chronic lymphocytic leukemia. J. Clin. Oncol..

[102] Bhattacharyya ND, Feng CG (2020). Regulation of T helper cell fate by TCR signal strength. Front. Immunol..

[103] Feske S (2009). ORAI1 and STIM1 deficiency in human and mice: roles of store-operated Ca2+ entry in the immune system and beyond. Immunol. Rev..

[104] Feske S, Picard C, Fischer A (2010). Immunodeficiency due to mutations in ORAI1 and STIM1. Clin. Immunol..

[105] Lian J (2018). ORAI1 mutations abolishing store-operated Ca^2+^ entry cause anhidrotic ectodermal dysplasia with immunodeficiency. J. Allergy Clin. Immunol..

[106] Lacruz RS, Feske S (2015). Diseases caused by mutations in ORAI1 and STIM1. Ann. N. Y. Acad. Sci..

[107] Cheng KT (2012). STIM1 and STIM2 protein deficiency in T lymphocytes underlies development of the exocrine gland autoimmune disease, Sjogren’s syndrome. Proc. Natl Acad. Sci. USA.

[108] Oh-Hora M (2008). Dual functions for the endoplasmic reticulum calcium sensors STIM1 and STIM2 in T cell activation and tolerance. Nat. Immunol..

[109] Picard C (2009). STIM1 mutation associated with a syndrome of immunodeficiency and autoimmunity. N. Engl. J. Med..

[110] Fuchs S (2012). Antiviral and regulatory T cell immunity in a patient with stromal interaction molecule 1 deficiency. J. Immunol..

[111] Srikanth S (2019). The Ca^2+^ sensor STIM1 regulates the type I interferon response by retaining the signaling adaptor STING at the endoplasmic reticulum. Nat. Immunol..

[112] Nunes-Santos CJ, Uzel G, Rosenzweig SD (2019). PI3K pathway defects leading to immunodeficiency and immune dysregulation. J. Allergy Clin. Immunol..

[113] Lucas CL (2014). Dominant-activating germline mutations in the gene encoding the PI(3)K catalytic subunit p110δ result in T cell senescence and human immunodeficiency. Nat. Immunol..

[114] Suzuki A (2001). T cell-specific loss of Pten leads to defects in central and peripheral tolerance. Immunity.

[115] Moser EK, Oliver PM (2019). Regulation of autoimmune disease by the E3 ubiquitin ligase Itch. Cell. Immunol..

[116] Teachey DT (2008). Targeting Notch signaling in autoimmune and lymphoproliferative disease. Blood.

[117] Shembade N (2008). The E3 ligase Itch negatively regulates inflammatory signaling pathways by controlling the function of the ubiquitin-editing enzyme A20. Nat. Immunol..

[118] Kleine-Eggebrecht N (2019). Mutation in *ITCH* Gene Can Cause Syndromic Multisystem Autoimmune Disease With Acute Liver Failure. Pediatrics.

[119] Lohr NJ (2010). Human ITCH E3 ubiquitin ligase deficiency causes syndromic multisystem autoimmune disease. *Am*. J. Hum. Genet..

[120] Keller B (2016). Early onset combined immunodeficiency and autoimmunity in patients with loss-of-function mutation in LAT. J. Exp. Med..

[121] Sommers CL (2002). A LAT mutation that inhibits T cell development yet induces lymphoproliferation. Science.

[122] Sommers CL (2005). Mutation of the phospholipase C-gamma1-binding site of LAT affects both positive and negative thymocyte selection. J. Exp. Med..

[123] Hauck F (2012). Primary T-cell immunodeficiency with immunodysregulation caused by autosomal recessive LCK deficiency. J. Allergy Clin. Immunol..

[124] Rowe JH (2018). Patients with *CD3G* mutations reveal a role for human CD3γ in T_reg_ diversity and suppressive function. Blood.

[125] Lee WI (2019). A Novel *CD3G* mutation in a taiwanese patient with normal t regulatory function presenting with the CVID phenotype free of autoimmunity-analysis of all genotypes and phenotypes. Front. Immunol..

[126] Schejbel L (2011). Molecular basis of hereditary C1q deficiency-revisited: identification of several novel disease-causing mutations. Genes Immun..

[127] Lipsker D, Hauptmann G (2010). Cutaneous manifestations of complement deficiencies. Lupus.

[128] Eisen DP, Minchinton RM (2003). Impact of mannose-binding lectin on susceptibility to infectious diseases. Clin. Infect. Dis..

[129] Tsutsumi A, Takahashi R, Sumida T (2005). Mannose binding lectin: genetics and autoimmune disease. Autoimmun. Rev..

[130] Pickering MC, Botto M, Taylor PR, Lachmann PJ, Walport MJ (2000). Systemic lupus erythematosus, complement deficiency, and apoptosis. Adv. Immunol..

[131] Stengaard-Pedersen K (2003). Inherited deficiency of mannan-binding lectin-associated serine protease 2. N. Engl. J. Med..

[132] Fleisher TA, Oliveira JB (2012). Monogenic defects in lymphocyte apoptosis. Curr. Opin. Allergy Clin. Immunol..

[133] Rieux-Laucat F, Le Deist F, Fischer A (2003). Autoimmune lymphoproliferative syndromes: genetic defects of apoptosis pathways. Cell Death Differ..

[134] Bride K, Teachey D (2017). Autoimmune lymphoproliferative syndrome: more than a FAScinating disease. F1000Res..

[135] Neven B (2011). A survey of 90 patients with autoimmune lymphoproliferative syndrome related to TNFRSF6 mutation. Blood.

[136] Molnár E (2020). Key diagnostic markers for autoimmune lymphoproliferative syndrome with molecular genetic diagnosis. Blood.

[137] Rensing-Ehl A (2010). Clinical and immunological overlap between autoimmune lymphoproliferative syndrome and common variable immunodeficiency. Clin. Immunol..

[138] Rieux-Laucat F, Fischer A, Deist FL (2003). Cell-death signaling and human disease. Curr. Opin. Immunol..

[139] Rieux-Laucat F (2006). Inherited and acquired death receptor defects in human Autoimmune Lymphoproliferative Syndrome. Curr. Dir. Autoimmun..

[140] Hsu AP (2012). Autoimmune lymphoproliferative syndrome due to FAS mutations outside the signal-transducing death domain: molecular mechanisms and clinical penetrance. Genet. Med..

[141] Hauck F (2013). Somatic loss of heterozygosity, but not haploinsufficiency alone, leads to full-blown autoimmune lymphoproliferative syndrome in 1 of 12 family members with FAS start codon mutation. Clin. Immunol..

[142] Magerus-Chatinet A (2011). Onset of autoimmune lymphoproliferative syndrome (ALPS) in humans as a consequence of genetic defect accumulation. J. Clin. Invest..

[143] Miano M (2019). FAS-mediated apoptosis impairment in patients with ALPS/ALPS-like phenotype carrying variants on CASP10 gene. Br. J. Haematol..

[144] Niemela JE (2011). Somatic KRAS mutations associated with a human nonmalignant syndrome of autoimmunity and abnormal leukocyte homeostasis. Blood.

[145] Oliveira JB (2007). NRAS mutation causes a human autoimmune lymphoproliferative syndrome. Proc. Natl Acad. Sci. USA.

[146] Crow MK, Olferiev M, Kirou KA, Type I (2019). Interferons in Autoimmune Disease. Annu. Rev. Pathol..

[147] Jiang, J. et al Interferons in the pathogenesis and treatment of autoimmune diseases. *Clin. Rev. Allergy Immunol*. 10.1007/s12016-020-08798-2 (2020). 10.1007/s12016-020-08798-2 32557263

[148] Bienias M (2018). Therapeutic approaches to Type I Interferonopathies. Curr. Rheumatol. Rep..

[149] Mogensen TH (2019). IRF and STAT transcription factors - from basic biology to roles in infection, protective immunity, and primary immunodeficiencies. Front. Immunol..

[150] Toubiana J (2016). Heterozygous STAT1 gain-of-function mutations underlie an unexpectedly broad clinical phenotype. Blood.

[151] Chen X (2020). Molecular and phenotypic characterization of nine patients with STAT1 GOF mutations in China. J. Clin. Immunol..

[152] Ovadia A, Sharfe N, Hawkins C, Laughlin S, Roifman CM (2018). Two different STAT1 gain-of-function mutations lead to diverse IFN-γ-mediated gene expression. NPJ Genom. Med..

[153] Rodero MP, Crow YJ (2016). Type I interferon-mediated monogenic autoinflammation: The type I interferonopathies, a conceptual overview. J. Exp. Med..

[154] Kaleviste E (2019). Interferon signature in patients with STAT1 gain-of-function mutation is epigenetically determined. Eur. J. Immunol..

[155] Zhou Q (2014). Early-onset stroke and vasculopathy associated with mutations in ADA2. N. Engl. J. Med..

[156] Navon Elkan P (2014). Mutant adenosine deaminase 2 in a polyarteritis nodosa vasculopathy. N. Engl. J. Med.

[157] Skrabl-Baumgartner A (2017). Autoimmune phenotype with type I interferon signature in two brothers with ADA2 deficiency carrying a novel CECR1 mutation. Pediatr. Rheumatol. Online J..

[158] Insalaco A (2019). Variable clinical phenotypes and relation of interferon signature with disease activity in ADA2 deficiency. J. Rheumatol..

[159] Cashman KS (2019). Understanding and measuring human B-cell tolerance and its breakdown in autoimmune disease. Immunol. Rev..

[160] Gay D, Saunders T, Camper S, Weigert M (1993). Receptor editing: an approach by autoreactive B cells to escape tolerance. J. Exp. Med..

[161] Tiegs SL, Russell DM, Nemazee D (1993). Receptor editing in self-reactive bone marrow B cells. J. Exp. Med..

[162] Kumar R, DiMenna LJ, Chaudhuri J, Evans T (2014). Biological function of activation-induced cytidine deaminase (AID). Biomed. J..

[163] Quartier P (2004). Clinical, immunologic and genetic analysis of 29 patients with autosomal recessive hyper-IgM syndrome due to Activation-Induced Cytidine Deaminase deficiency. Clin. Immunol..

[164] Revy P (2000). Activation-induced cytidine deaminase (AID) deficiency causes the autosomal recessive form of the Hyper-IgM syndrome (HIGM2). Cell.

[165] Meyers G (2011). Activation-induced cytidine deaminase (AID) is required for B-cell tolerance in humans. Proc. Natl Acad. Sci. USA.

[166] Mackay F (1999). Mice transgenic for BAFF develop lymphocytic disorders along with autoimmune manifestations. J. Exp. Med..

[167] Qin H (2011). Activation-induced cytidine deaminase expression in CD4+ T cells is associated with a unique IL-10-producing subset that increases with age. PLoS One.

[168] Durandy A, Cantaert T, Kracker S, Meffre E (2013). Potential roles of activation-induced cytidine deaminase in promotion or prevention of autoimmunity in humans. Autoimmunity.

[169] Salzer E, Santos-Valente E, Keller B, Warnatz K, Boztug K (2016). Protein Kinase C δ: a Gatekeeper of Immune Homeostasis. J. Clin. Immunol..

[170] Salzer E (2013). B-cell deficiency and severe autoimmunity caused by deficiency of protein kinase C δ. Blood.

[171] Belot A (2013). Protein kinase cδ deficiency causes mendelian systemic lupus erythematosus with B cell-defective apoptosis and hyperproliferation. Arthritis Rheum..

[172] Kuehn HS (2013). Loss-of-function of the protein kinase C δ (PKCδ) causes a B-cell lymphoproliferative syndrome in humans. Blood.

[173] Kiykim A (2015). Potentially beneficial effect of hydroxychloroquine in a patient with a novel mutation in protein kinase Cδ deficiency. J. Clin. Immunol..

[174] Miyamoto A (2002). Increased proliferation of B cells and auto-immunity in mice lacking protein kinase Cdelta. Nature.

[175] Ehl S, Hombach J, Aichele P, Hengartner H, Zinkernagel RM (1997). Bystander activation of cytotoxic T cells: studies on the mechanism and evaluation of in vivo significance in a transgenic mouse model. J. Exp. Med..

[176] Llewelyn M, Cohen J (2002). Superantigens: microbial agents that corrupt immunity. Lancet Infect. Dis..

[177] Fujinami RS, von Herrath MG, Christen U, Whitton JL (2016). Molecular mimicry, bystander activation, or viral persistence: infections and autoimmune disease. Clin. Microbiol. Rev..

[178] Hussein HM, Rahal EA (2019). The role of viral infections in the development of autoimmune diseases. Crit. Rev. Microbiol..

[179] Rodríguez Y (2018). Guillain-Barré syndrome, transverse myelitis and infectious diseases. Cell. Mol. Immunol..

[180] Qiu CC, Caricchio R, Gallucci S (2019). Triggers of autoimmunity: the role of bacterial infections in the extracellular exposure of lupus nuclear autoantigens. Front. Immunol..

[181] Christen U, von Herrath MG (2005). Infections and autoimmunity-good or bad?. J. Immunol..

[182] Draborg AH, Duus K, Houen G (2012). Epstein-Barr virus and systemic lupus erythematosus. Clin. Dev. Immunol..

[183] Newkirk MM, Zbar A, Baron M, Manges AR (2010). Distinct bacterial colonization patterns of Escherichia coli subtypes associate with rheumatoid factor status in early inflammatory arthritis. Rheumatol. (Oxf.).

[184] Kivity S, Agmon-Levin N, Blank M, Shoenfeld Y (2009). Infections and autoimmunity-friends or foes?. Trends Immunol..

[185] Rodriguez-Calvo T (2019). Enterovirus infection and type 1 diabetes: unraveling the crime scene. Clin. Exp. Immunol..

[186] Nakamura H, Shimizu T, Kawakami A (2020). Role of Viral Infections in the Pathogenesis of Sjögren’s Syndrome: Different Characteristics of Epstein-Barr Virus and HTLV-1. J. Clin. Med..

[187] Venigalla SSK, Premakumar S, Janakiraman V (2020). A possible role for autoimmunity through molecular mimicry in alphavirus mediated arthritis. Sci. Rep..

[188] Dow CT, Proposing BCG (2020). Vaccination for *Mycobacterium avium* ss. *paratuberculosis* (MAP) Associated Autoimmune Diseases. Microorganisms.

[189] Sogkas G (2020). CD74 is a T cell antigen in spondyloarthritis. Clin. Exp. Rheumatol..

[190] Cusick MF, Libbey JE, Fujinami RS (2012). Molecular mimicry as a mechanism of autoimmune disease. Clin. Rev. Allergy Immunol..

[191] Dematapitiya C (2019). Cold type autoimmune hemolytic anemia- a rare manifestation of infectious mononucleosis; serum ferritin as an important biomarker. BMC Infect. Dis..

[192] Fadeyi EA, Simmons JH, Jones MR, Palavecino EL, Pomper GJ (2015). Fatal autoimmune hemolytic anemia due to immunoglobulin g autoantibody exacerbated by epstein-barr virus. Lab. Med..

[193] Kang I (2004). Defective control of latent Epstein-Barr virus infection in systemic lupus erythematosus. J. Immunol..

[194] Larsen M (2011). Exhausted cytotoxic control of Epstein-Barr virus in human lupus. PLoS Pathog..

[195] Pender MP (2009). Preventing and curing multiple sclerosis by controlling Epstein-Barr virus infection. Autoimmun. Rev..

[196] Klatt T (2005). Expansion of peripheral CD8+ CD28- T cells in response to Epstein-Barr virus in patients with rheumatoid arthritis. J. Rheumatol..

[197] Cohen JI (2015). Primary immunodeficiencies associated with EBV disease. Curr. Top. Microbiol. Immunol..

[198] Latour S, Winter S (2018). Inherited immunodeficiencies with high predisposition to epstein-barr virus-driven lymphoproliferative diseases. Front. Immunol..

[199] Ghosh S, Bienemann K, Boztug K, Borkhardt A (2014). Interleukin-2-inducible T-cell kinase (ITK) deficiency - clinical and molecular aspects. J. Clin. Immunol..

[200] Ravell J, Chaigne-Delalande B, Lenardo M (2014). X-linked immunodeficiency with magnesium defect, Epstein-Barr virus infection, and neoplasia disease: a combined immune deficiency with magnesium defect. Curr. Opin. Pediatr..

[201] Machado Ribeiro F, Goldenberg T (2015). Mycobacteria and autoimmunity. Lupus.

[202] Kakumanu P (2008). Patients with pulmonary tuberculosis are frequently positive for anti-cyclic citrullinated peptide antibodies, but their sera also react with unmodified arginine-containing peptide. Arthritis Rheum..

[203] Esaguy N, Aguas AP, van Embden JD, Silva MT (1991). Mycobacteria and human autoimmune disease: direct evidence of cross-reactivity between human lactoferrin and the 65-kilodalton protein of tubercle and leprosy bacilli. Infect. Immun..

[204] Mok MY (2007). Non-tuberculous mycobacterial infection in patients with systemic lupus erythematosus. Rheumatol. (Oxf.).

[205] Chao WC (2017). Association between a history of mycobacterial infection and the risk of newly diagnosed Sjögren’s syndrome: A nationwide, population-based case-control study. PLoS One.

[206] Rosain J (2019). Mendelian susceptibility to mycobacterial disease: 2014-2018 update. Immunol. Cell. Biol..

[207] Jindal AK (2019). Recurrent salmonella typhi infection and autoimmunity in a young boy with complete IL-12 receptor β1 deficiency. J. Clin. Immunol..

[208] Sogkas G (2017). First association of interleukin 12 receptor beta 1 deficiency with Sjögren’s syndrome. Front. Immunol..

[209] Göktürk B (2016). Infectious diseases, autoimmunity and midline defect in a patient with a novel bi-allelic mutation in IL12RB1 gene. Turk. J. Pediatr..

[210] Ling G (2016). IL-12 receptor 1β deficiency with features of autoimmunity and photosensitivity. Autoimmunity.

[211] Miraghazadeh B, Cook MC (2018). Nuclear factor-kappaB in autoimmunity: man and mouse. Front. Immunol..

[212] Tuijnenburg P (2018). Loss-of-function nuclear factor κB subunit 1 (NFKB1) variants are the most common monogenic cause of common variable immunodeficiency in Europeans. J. Allergy Clin. Immunol..

[213] Schröder C (2019). Late-onset antibody deficiency due to monoallelic alterations in *NFKB1*. Front. Immunol..

[214] Lorenzini, T. et al. Characterization of the clinical and immunologic phenotype and management of 157 individuals with 56 distinct heterozygous NFKB1 mutations. *J. Allergy Clin. Immunol*. S0091–6749(20)30422-X (2020). 10.1016/j.jaci.2019.11.051 PMC8246418 32278790

[215] Boisson B, Puel A, Picard C, Casanova JL (2017). Human IκBα Gain of Function: a Severe and Syndromic Immunodeficiency. J. Clin. Immunol..

[216] Sogkas G (2020). A novel NFKBIA variant substituting serine 36 of IκBα causes immunodeficiency with warts, bronchiectasis and juvenile rheumatoid arthritis in the absence of ectodermal dysplasia. Clin. Immunol..

[217] Batlle-Masó L (2020). Genetic diagnosis of autoinflammatory disease patients using clinical exome sequencing. Eur. J. Med. Genet..

[218] Seghezzo SP, Dvorak CC, Cowan MJ, Puck JM, Dorsey MJ (2020). Extended Follow-up After Hematopoietic Cell Transplantation for IκBα Deficiency with Disseminated Mycobacterium avium Infection. J. Clin. Immunol..

[219] Tan, E. E. et al. Dominant-negative NFKBIA mutation promotes IL-1β production causing hepatic disease with severe immunodeficiency. *J. Clin. Invest.* **130**, 5817–5832 (2020). 10.1172/JCI98882 PMC7598087 32750042

[220] Chang JH (2012). Ubc13 maintains the suppressive function of regulatory T cells and prevents their conversion into effector-like T cells. Nat. Immunol..

[221] Sun SC, Chang JH, Jin J (2013). Regulation of nuclear factor-κB in autoimmunity. Trends Immunol..

[222] Kaustio M (2017). Damaging heterozygous mutations in NFKB1 lead to diverse immunologic phenotypes. J. Allergy Clin. Immunol..

[223] Vasanthakumar A (2017). The TNF receptor superfamily-NF-κB axis is critical to maintain effector regulatory T cells in lymphoid and non-lymphoid tissues. Cell. Rep..

[224] Kollias G (2005). TNF pathophysiology in murine models of chronic inflammation and autoimmunity. Semin. Arthritis Rheum..

[225] Janssen R (2004). The same IkappaBalpha mutation in two related individuals leads to completely different clinical syndromes. J. Exp. Med..

[226] Lu HY (2019). Germline CBM-opathies: from immunodeficiency to atopy. J. Allergy Clin. Immunol..

[227] Gupta M (2018). Clinical, immunological, and molecular findings in four cases of B cell expansion with NF-κB and T cell anergy disease for the first time from India. Front. Immunol..

[228] Bedsaul JR (2018). Mechanisms of regulated and dysregulated CARD11 signaling in adaptive immunity and disease. Front. Immunol..

[229] Dorjbal B (2019). Hypomorphic caspase activation and recruitment domain 11 (CARD11) mutations associated with diverse immunologic phenotypes with or without atopic disease. J. Allergy Clin. Immunol..

[230] Fuchs S (2015). Omenn syndrome associated with a functional reversion due to a somatic second-site mutation in CARD11 deficiency. Blood.

[231] Charbit-Henrion F (2017). Deficiency in Mucosa-associated Lymphoid Tissue Lymphoma Translocation 1: A Novel Cause of IPEX-Like Syndrome. J. Pediatr. Gastroenterol. Nutr..

[232] Bornancin F (2015). Deficiency of MALT1 paracaspase activity results in unbalanced regulatory and effector T and B cell responses leading to multiorgan inflammation. J. Immunol..

[233] Torres JM (2014). Inherited BCL10 deficiency impairs hematopoietic and nonhematopoietic immunity. J. Clin. Invest..

[234] Jablonka A (2020). Peripheral blood lymphocyte phenotype differentiates secondary antibody deficiency in rheumatic disease from primary antibody deficiency. J. Clin. Med..

[235] Fessler, J. et al. Lymphopenia in primary Sjögren’s syndrome is associated with premature aging of naïve CD4+ T cells. *Rheumatology (Oxford)* keaa105 (2020). 10.1093/rheumatology/keaa105 32227243

[236] Martin M, Guffroy A, Argemi X, Martin T (2017). [Systemic lupus erythematosus and lymphopenia: Clinical and pathophysiological features]. Rev. Med. Interne.

[237] Du J (2017). The association between the lymphocyte-monocyte ratio and disease activity in rheumatoid arthritis. Clin. Rheumatol..

[238] Okada Y (2014). Genetics of rheumatoid arthritis contributes to biology and drug discovery. Nature.

[239] Chopra C (2014). Immune deficiency in Ataxia-Telangiectasia: a longitudinal study of 44 patients. Clin. Exp. Immunol..

[240] McAllister K (2013). Identification of BACH2 and RAD51B as rheumatoid arthritis susceptibility loci in a meta-analysis of genome-wide data. Arthritis Rheum..

[241] Morris DL (2016). Genome-wide association meta-analysis in Chinese and European individuals identifies ten new loci associated with systemic lupus erythematosus. Nat. Genet..

[242] Plenge RM (2005). Replication of putative candidate-gene associations with rheumatoid arthritis in >4,000 samples from North America and Sweden: association of susceptibility with PTPN22, CTLA4, and PADI4. Am. J. Hum. Genet..

[243] Sun C (2016). High-density genotyping of immune-related loci identifies new SLE risk variants in individuals with Asian ancestry. Nat. Genet..

[244] Barton A (2008). Rheumatoid arthritis susceptibility loci at chromosomes 10p15, 12q13 and 22q13. Nat. Genet.

[245] Tanaka Y (1999). Association of the interferon-gamma receptor variant (Val14Met) with systemic lupus erythematosus. Immunogenetics.

[246] Kong XF (2013). Haploinsufficiency at the human IFNGR2 locus contributes to mycobacterial disease. Hum. Mol. Genet..

[247] Moncada-Vélez M (2013). Partial IFN-γR2 deficiency is due to protein misfolding and can be rescued by inhibitors of glycosylation. Blood.

[248] Wei WH, Viatte S, Merriman TR, Barton A, Worthington J (2017). Genotypic variability based association identifies novel non-additive loci DHCR7 and IRF4 in sero-negative rheumatoid arthritis. Sci. Rep..

[249] Fu Q (2011). Association of a functional IRF7 variant with systemic lupus erythematosus. Arthritis Rheum..

[250] Ciancanelli MJ (2015). Infectious disease. Life-threatening influenza and impaired interferon amplification in human IRF7 deficiency. Science.

[251] Yokoyama N (2019). Association of NCF1 polymorphism with systemic lupus erythematosus and systemic sclerosis but not with ANCA-associated vasculitis in a Japanese population. Sci. Rep..

[252] Zhao J (2017). A missense variant in NCF1 is associated with susceptibility to multiple autoimmune diseases. Nat. Genet..

[253] Yu, H.H., Yang, Y.H. & Chiang, B.L. Chronic granulomatous disease: a comprehensive review. *Clin. Rev. Allergy Immunol*. 10.1007/s12016-020-08800-x (2020). 10.1007/s12016-020-08800-x 32524254

[254] Tsoi LC (2012). Identification of 15 new psoriasis susceptibility loci highlights the role of innate immunity. Nat. Genet..

[255] Papageorgiou A (2015). A BAFF receptor His159Tyr mutation in Sjögren’s syndrome-related lymphoproliferation. Arthritis Rheumatol..

[256] Warnatz K (2019). B-cell activating factor receptor deficiency is associated with an adult-onset antibody deficiency syndrome in humans. Proc. Natl Acad. Sci. USA.

[257] Cortes A (2013). Identification of multiple risk variants for ankylosing spondylitis through high-density genotyping of immune-related loci. Nat. Genet..

[258] Kreins AY (2015). Human TYK2 deficiency: Mycobacterial and viral infections without hyper-IgE syndrome. J. Exp. Med..

[259] Oliveira JB (2010). Revised diagnostic criteria and classification for the autoimmune lymphoproliferative syndrome (ALPS): report from the 2009 NIH International Workshop. Blood.

[260] Crow YJ, Shetty J, Livingston JH (2020). Treatments in Aicardi-Goutières syndrome. Dev. Med. Child Neurol..

[261] Lin B (2020). A novel STING1 variant causes a recessive form of STING-associated vasculopathy with onset in infancy (SAVI). J. Allergy Clin. Immunol..

[262] Fiehn C (2017). Familial Chilblain Lupus - what can we learn from Type I interferonopathies?. Curr. Rheumatol. Rep..

[263] Briggs, T. A. et al. Spondyloenchondrodysplasia due to mutations in ACP5: a comprehensive survey. *J. Clin. Immunol.* **36**, 220–234 10.1007/s10875-016-0252-y PMC4792361 26951490

[264] Torrelo A (2017). CANDLE syndrome as a paradigm of proteasome-related autoinflammation. Front. Immunol..

[265] Saito R (2019). Retinal vasculopathy with cerebral leukodystrophy: clinicopathologic features of an autopsied patient with a heterozygous TREX 1 mutation. J. Neuropathol. Exp. Neurol..

[266] Rice GI (2020). Genetic and phenotypic spectrum associated with IFIH1 gain-of-function. Hum. Mutat..

[267] Lässig C (2018). Unified mechanisms for self-RNA recognition by RIG-I Singleton-Merten syndrome variants. Elife.

[268] Martin-Fernandez M (2020). Systemic Type I IFN inflammation in human ISG15 deficiency leads to necrotizing skin lesions. Cell. Rep..

[269] Meuwissen ME (2016). Human USP18 deficiency underlies type 1 interferonopathy leading to severe pseudo-TORCH syndrome. J. Exp. Med..

[270] Van Esch H (2019). Defective DNA polymerase α-primase leads to X-linked intellectual disability associated with severe growth retardation, microcephaly, and hypogonadism. Am. J. Hum. Genet..

[271] Rodero MP (2017). Type I interferon-mediated autoinflammation due to DNase II deficiency. Nat. Commun..

